# Structural insights into the exchange mechanism of a replicative DNA polymerase

**DOI:** 10.1093/nar/gkaf1359

**Published:** 2025-12-29

**Authors:** Xiang Feng, Michelle M Spiering, Almeida Ruda de Luna Santos, Stephen J Benkovic, Huilin Li

**Affiliations:** Department of Structural Biology, Van Andel Institute, Grand Rapids, MI 49503, United States; Department of Chemistry, The Pennsylvania State University, University Park, PA 16802, United States; Department of Structural Biology, Van Andel Institute, Grand Rapids, MI 49503, United States; Department of Chemistry, The Pennsylvania State University, University Park, PA 16802, United States; Department of Structural Biology, Van Andel Institute, Grand Rapids, MI 49503, United States

## Abstract

Replicative DNA polymerases are distinguished by their speed, processivity, and fidelity. While speed and fidelity arise from the polymerase’s intrinsic catalytic and proofreading activities, processivity is typically attributed to the DNA sliding clamp that tethers the polymerase to DNA. However, additional mechanisms may also contribute. The T4 bacteriophage polymerase can exchange on-the-fly, a process likely contributing to its ∼10-fold higher synthesis rate compared with human polymerases. Here, we reconstituted the T4 holoenzyme and polymerase exchange complexes using purified gp43 polymerase, gp45 sliding clamp, and a primer–template DNA substrate. Cryo-electron microscopy (cryo-EM) analysis revealed either one or two polymerases bound to the clamp and DNA. In the one-polymerase complex, the DNA threads perpendicularly through the clamp, supporting processive synthesis. In contrast, the two-polymerase complex displays a markedly tilted DNA orientation, impeding sliding and representing exchange intermediates. Three distinct conformational states of the two-polymerase complex define a multistep exchange mechanism. To our knowledge, these findings provide the first molecular-level view of replicative polymerase exchange.

## Graphical Abstract

**Graphical Abstract:**
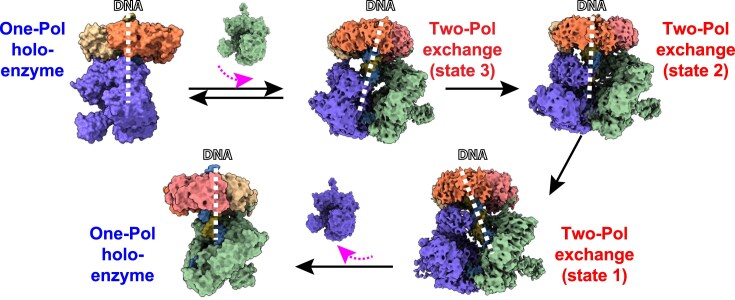

## Introduction

Since the 1940s, the T4 bacteriophage has served as an important model for studying DNA replication and repair mechanisms. T4 phage demonstrates remarkable efficiency and fidelity in DNA replication. The replication machinery operates at 500–750 nucleotides per second [1]—significantly faster than the ∼50 nucleotides per second observed in human cells [2, 3]—while maintaining exceptional accuracy with a mutation rate of just 2 × 10⁻⁸ per base pair [4, 5]. With only eight gene products comprising its DNA replication machinery, T4 provides a relatively straightforward system that shares key features with more complex replication processes, making it valuable for understanding fundamental principles of DNA replication and repair.

The T4 replication system centers on a holoenzyme complex that is assembled from the DNA polymerase (gp43) and the sliding clamp (gp45) loaded onto a primer/template junction by the clamp loader complex (gp44/62) in the presence of ATP [6, 7]. However, laboratory studies have shown that the clamp loader isn’t strictly necessary for holoenzyme assembly when working with short DNA substrates, as the clamp can bind directly to duplex DNA ends and load itself [8]. The sliding clamp plays a crucial role by encircling the DNA double helix and anchoring the polymerase to the template. This attachment enables the rapid, continuous DNA synthesis characteristic of T4 replication. Without the clamp, the gp43 polymerase tends to dissociate frequently from the DNA, resulting in much slower and less efficient synthesis [9]. This sliding clamp and clamp loader system represents an evolutionarily conserved mechanism found across all domains of life, highlighting its fundamental importance for achieving high-speed, processive DNA replication [10].

**Figure 1.**
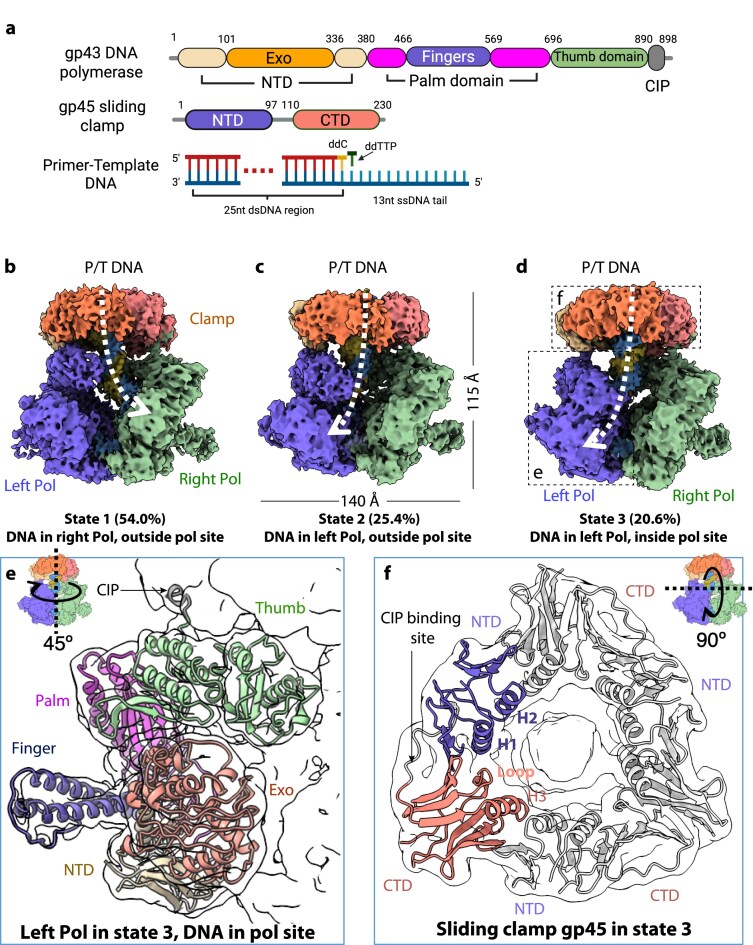
Cryo-EM captures a T4 polymerase exchange complex containing two polymerases. (**A**) Domain organization of the gp43 polymerase (Pol), gp45 clamp, and P/T DNA substrate. Created in BioRender, https://BioRender.com/bfkkkot. (**B–D**) Electron microscopy (EM) maps of the two-polymerase complex in three conformational states [1–3], distinguished by the DNA trajectory (dashed white curves). The relative population of each state is indicated. (**E**) Tilted side view (45°) highlighting the left polymerase in state 3. (**F**) Top view of the gp45 clamp. In panels (E) and (F), the atomic models (cartoon representation) are fitted into the transparent surface-rendered EM maps. Major protein domains are labeled.

The gp43 polymerase is classified within the B-family of DNA polymerases, sharing this grouping with *Escherichia coli* Pol II and the gp43 polymerase from RB69 phage. Sequence analysis reveals that T4 polymerase contains three main structural regions: an N-terminal domain, a 3′-5′ exonuclease domain responsible for proofreading activity, and a C-terminal polymerase domain. The polymerase domain adopts the characteristic “right hand” architecture common to DNA polymerases, featuring distinct subregions known as the palm, fingers, and thumb [11] (Fig. 1A). While the complete structure of T4 polymerase remains undetermined except for an N-terminal fragment (residues 1–369) [12], detailed crystal structures are available for the closely related RB69 gp43 polymerase (reviewed in [13]). With 62% sequence identity to T4 gp43, RB69 polymerase serves as an excellent structural model for understanding T4 polymerase function.

In the RB69 structure, the thumb subdomain plays a critical role in directing the primer’s 3′-terminus to the appropriate active site depending on the polymerase’s operational mode. During proofreading, the thumb guides the primer terminus to the exonuclease active site for removal of incorrectly incorporated nucleotides [14]. Conversely, during DNA synthesis, it directs the primer to the polymerization active site located within the finger and palm subdomains [15]. The finger domain exhibits dynamic behavior, opening to allow nucleotide binding and exchange, then closing to properly position and stabilize the incoming dNTP at the polymerization site [13].

An important feature of the T4 polymerase is a clamp interacting peptide (CIP) motif located in its C-terminal region, which is essential for holoenzyme formation. Deletion of the last six C-terminal amino acids prevents the T4 polymerase from associating with the sliding clamp [16], thereby impairing its processivity. This CIP motif is widely conserved among clamp-interacting proteins in both phages and bacteria [17–19], and shows structural similarity to the proliferating cell nuclear antigen (PCNA)-interacting peptide motif found in eukaryotic systems [20], highlighting the evolutionary conservation of polymerase–clamp interactions across different domains of life.

Sliding clamps represent a highly conserved ring-shaped protein architecture found across all domains of life, from clamps in bacteriophages [14, 21] to β-clamps in bacteria [22] to PCNA in eukaryotic organisms like yeast and humans [23]. The T4 gp45 sliding clamp forms a trimeric ring structure, with each of the three monomers composed of structurally similar N-terminal and C-terminal domains (NTD and CTD). Each domain contains a characteristic β–α–β–β–β structural motif that appears twice, creating a twofold repeat pattern. This arrangement generates a ring encircled by antiparallel β-sheets, while the central DNA-binding channel is typically lined by 12 α-helices (designated H1-4, contributed by each of the three subunits) [24]. Early crystal structural studies suggested that DNA passes through the clamp at an angle, with direct physical contact between the DNA backbone and the clamp’s inner channel surface [25, 26]. However, more recent structural analyses of sliding clamps bound to B-family polymerases have challenged this model. These newer structures reveal that the DNA orients perpendicular to the clamp plane, and importantly, a layer of water molecules exists between the DNA and the clamp channel walls. This discovery has led to the “water skating” hypothesis, proposing that the clamp glides along the DNA backbone on a thin film of water molecules during the DNA synthesis process, rather than making direct contact with the DNA substrate [27–29].

In the T4 replisome, both the continuously synthesized leading strand and the discontinuously synthesized lagging strand are replicated by gp43 DNA polymerases in separate holoenzyme complexes with gp45 sliding clamps. The leading-strand holoenzyme demonstrates remarkable processivity, maintaining an ∼11-min half-life while the replication fork advances [30]. Alberts and coworkers originally proposed that lagging-strand polymerase persistence at the fork occurs through polymerase dimerization, enabling rapid and efficient recycling of the polymerase to successive RNA primers following completion of each Okazaki fragment [31]. Strong evidence supporting coordination between the leading- and lagging-strand holoenzyme assemblies derives from observations that DNA synthesis on both strands continues unimpaired when reaction components are diluted [32], indicating highly processive replication for both strands. However, this processive DNA synthesis on either the leading or lagging strands can be interrupted by a catalytically defective D408N gp43 mutant, which retains wild-type DNA binding affinity and clamp association [33]. The effect of this polymerase trap reveals dynamic polymerase processivity during replication—specifically, that polymerases can exchange between solution and the replisome without disrupting ongoing DNA synthesis. The molecular basis of this exchange mechanism remains unclear.

We conducted a comprehensive cryo-EM structural investigation of T4 polymerase complexes assembled *in vitro* with sliding clamps and primer/template (P/T) DNA substrates. Our analysis revealed two distinct complex types: a replicative holoenzyme containing a single polymerase bound to the sliding clamp and DNA, and a polymerase exchange assembly featuring two polymerases concurrently associated with the clamp and DNA. Remarkably, the two-polymerase complex was captured in three distinct conformational states. These structural snapshots allowed us to trace the stepwise process through which one polymerase replaces another, providing unprecedented insight into a multi-step polymerase exchange mechanism. We disrupted the polymerase dimer interface through targeted mutagenesis, creating an enzyme capable of forming only one-polymerase complexes. Importantly, these mutations did not affect DNA synthesis rates, demonstrating that this specific dimer interface is exclusively involved in the polymerase exchange process.

## Materials and methods

### Mutagenesis, expression, and purification of the T4 proteins

The construction of expression plasmids for wt-gp45 clamp [34] and gp43(exo-) exonuclease-deficient polymerase [35] were described previously. The dimer-interface mutant polymerase was generated by introducing three site-directed mutations D75R, Q430E, and K432E into the gp43(exo-)-intein fusion pET-IMPACT vector. The mutations were made using the QuikChange Lightning Multi Site-Directed Mutagenesis Kit (Agilent) with the D75R mutagenic forward primer 5′-GAA GCG AAT GGA ACG CAT CGG TCT CG-3′ and the Q430E, and K432E double mutagenic forward primer 5′-CGT GGT GAG TTT GAA GTT CAT CCA ATT C-3′ (the boldface underlined letters indicate the mutation sites).

The pET26b expression plasmid for gp45 clamp was transformed into *E. coli* BL21(DE3) cells and grown in NZCYM media at 37°C to an optical density of 0.4 at 600 nm. The cultures were then cooled to 18°C, and protein expression was induced with 0.4 mM isopropyl 1-thio-β-D-galactopyranoside. After 16–20 h of shaking, cells were harvested by centrifugation and resuspended in hydroxyapatite (HA) buffer A [20 mM potassium phosphate, pH 7.0, 5 mM magnesium sulfate, 1 mM ethylenediaminetetraacetic acid (EDTA), 10 mM β-mercaptoethanol, and 5% glycerol] with a cocktail of protease inhibitors (Roche). Cells were lysed using sonication and cell debris was pelleted at 40 000 × *g*. Nucleic acids in the cell-free extract were precipitated with the slow addition of ∼1% (final concentration) streptomycin sulfate while gently stirring at 4°C for 30 min followed by centrifugation at 20 000 × *g*. The supernatant was loaded onto a CHT Ceramic HA, Type II column (Bio-Rad) for cation exchange chromatography developed with a linear gradient of HA buffer B (250 mM potassium phosphate, pH 7.0, 5 mM magnesium sulfate, 1 mM EDTA, 10 mM β-mercaptoethanol, and 5% glycerol). The eluted protein was diluted into low salt buffer (20 mM TrisOAc, pH 7.8, 100 mM NaOAc, 0.1 mM EDTA, and 10% glycerol) for anion exchange chromatography developed with a linear gradient of high salt buffer (20 mM TrisOAc, pH 7.8, 500 mM NaOAc, 0.1 mM EDTA, and 10% glycerol). The eluted protein was dialyzed into storage buffer [10 mM TrisOAc, pH 7.8, 25 mM KOAc, 5 mM Mg(OAc)_2_, 2 mM dithiothreitol, and 20% glycerol] and analyzed for purity using sodium dodecyl sulphate–polyacrylamide gel electrophoresis (SDS–PAGE). Protein concentrations were determined by measuring the absorbance at 280 nm using an extinction coefficient for a homotrimer based on the protein sequence.

The self-cleaving, intein-based expression plasmids for gp43(exo-) and the dimer-interface mutant gp43(exo-) polymerase were transformed into *E. coli* BL21(DE3) cells and grown in NZCYM media at 37°C to an optical density of 0.4 at 600 nm. The cultures were then cooled to 18°C, and protein expression was induced with 0.4 mM isopropyl 1-thio-β-D-galactopyranoside. After 16–20 h of shaking, cells were harvested by centrifugation and resuspended in chitin column binding/high salt buffer (20 mM TrisOAc, pH 7.8, 1 M NaOAc, 0.1 mM EDTA, and 10% glycerol) with a cocktail of protease inhibitors (Roche). Cells were lysed using sonication and cell debris was pelleted at 40 000 × *g*. Cell-free extract was loaded onto chitin resin (New England Biolabs) for chitin-based affinity chromatography, and the chitin resin was washed with chitin column binding buffer. The resin was resuspended in cleavage/low salt buffer (20 mM TrisOAc, pH 7.8, 100 mM NaOAc, 0.1 mM EDTA, and 10% glycerol) with 75 mM β-mercaptoethanol and incubated overnight at 4°C to facilitate intein-mediated cleavage. The protein was eluted from the chitin column in low salt buffer for heparin-based affinity chromatography developed with a linear gradient of high salt buffer. The eluted protein was dialyzed into storage buffer [10 mM TrisOAc, pH 7.8, 25 mM KOAc, 5 mM Mg(OAc)_2_, 2 mM dithiothreitol, and 20% glycerol] and analyzed for purity using SDS–PAGE. Protein concentrations were determined by measuring the absorbance at 280 nm using extinction coefficients based on the protein sequence. Proteins for cryo-EM were purified in buffers containing HEPES (pH 7.8) instead of TrisOAc (pH 7.8) and were frozen for storage immediately following heparin-based affinity chromatography without dialysis into storage buffer.

### Holoenzyme assembly assay

All DNA oligos were purchased from Integrated DNA Technologies and PAGE purified. A single-stranded DNA (ssDNA) oligo 5′-/5Biosg/CTG CAC GAA TTA AGC AAT TCG TAA TCA TGG TCA TAG CT/3Bio/-3′ served as the template strand; neutravidin bound to the biotin on both ends created blocks to prevent assembled holoenzymes from sliding off the ends of the P/T DNA substrate. The complementary primer oligo 5′-AGC TAT GAC CAT GAT TAC GAA TTG C-3′ was radiolabeled at the 5′-end with T4 Polynucleotide Kinase and [γ-^32^P]ATP. The template (in 10% excess) and primer were annealed together by heating to 65°C for 30 min and slowly cooling to room temperature.

The stoichiometry and stability of the T4 holoenzymes were determined in replication buffer [25 mM TrisOAc, pH 7.8, 150 mM KOAc, and 10 mM Mg(OAc)_2_] at room temperature. Holoenzymes were assembled on P/T DNA for 30 s by incubating 500 nM P/T DNA, 1.1 μM neutravidin, and 1 mM ATP with 100 nM T4 polymerase (exo- or dimer interface mutant), 550 nM gp45 clamp (homotrimer) and 550 nM gp44/62 clamp loader. To prevent the formation of new holoenzymes, 1 mg/ml of ssDNA trap was added. DNA synthesis by any assembled complexes was initiated with the addition of 100 μM dNTPs after varying incubation times (from 30 s to 15 min). Reactions were quenched after 20 s by adding 83.3 μM EDTA and 1.6 units proteinase K. The quenched samples were incubated at 37°C for >5 min to allow proteinase K to act, then diluted 1:2 with formamide, 1 μg/ml each of bromophenol blue and xylene cyanol FF. Initial primers and extended products were separated using 16% denaturing urea-PAGE in 1× Tris–Borate–EDTA (TBE) buffer and analyzed with a phosphorimager. The migration position of the primer (25 nt) and the fully extended primer (38 nt) was verified by separately radiolabeling the primer and nonbiotinylated template and running them in separate lanes alongside the reaction products on the gel.

### Rolling circle assays

The rolling circle replication reactions were performed in the standard replication buffer containing 10 nM primed ssM13 DNA; 200 nM each gp45 trimeric clamp, gp44/62 clamp loader, and gp43 (either exo- or dimer interface mutant); 600 nM each gp59 helicase loader, gp41 helicase, and gp61 primase; 4 μM gp32; 200 μM dNTPs and rNTPs with 10 μCi of [α-32P]dGTP; and 2 mM ATP in a reaction volume of 25 μl. Time points (5 μl) were withdrawn and quenched after 1, 2.5, 5, and 10 min by adding an equal volume of alkaline stop buffer (100 mM NaOH, 2 mM EDTA, 60% Ficoll 400, 0.5 mg/ml bromocresol green, and 0.8 mg/ml xylene cyanol FF). The resulting DNA products were separated using 0.8% alkaline-agarose gel electrophoresis (in 50 mM NaOH and 1 mM EDTA) at 40 volts for 20 h. After electrophoresis, the gel was neutralized by soaking in 1× TBE buffer and then dried onto a sheet of DE81 paper, first using a stack of paper towels and subsequently with a vacuum gel-dryer. The dried gel and DE81 filter paper were analyzed using a phosphorimager.

### Cryo-EM grid preparation and data collection

All DNA oligos were purchased from Integrated DNA Technologies and either polyacrylamide gel electrophoresis (PAGE) or high performance liquid chromatography purified. A ssDNA oligo 5′-CTG CAC GAA TTA AGC AAT TCG TAA TCA TGG TCA TAG CT-3′ served as the template strand. The complementary primer oligo 5′-AGC TAT GAC CAT GAT TAC GAA TTG ddC-3′ contained a chain-terminating dideoxycytidine to prevent the polymerase from incorporating additional nucleotides. The template and primer were annealed together by heating to 90°C and slowly cooling to 4°C.

The polymerase complexes were reconstituted *in vitro* following previous protocols utilized for another B-family polymerase [28]. We first loaded the sliding clamp onto the primer/template DNA substrate by mixing 10 μM purified gp45 clamp protein with 5 μM P/T DNA in reaction buffer (20 mM HEPES, pH 7.5, 150 mM KOAc, 10 mM MgCl_2_, 0.5 mM ddTTP, and 2 mM dithiothreitol) for 10 min at 30°C. Then the polymerase complexes were formed by adding 6 μM gp43 polymerase and incubating the mixture at room temperature for 10 min followed by 1 h on ice. Aliquots (4 μl) of the mixtures were applied to glow-discharged holey carbon grids (Quantifoil R2/1 Copper, 300 mesh) in the humidity-controlled chamber of a FEI Vitrobot Mark IV. The EM grids were blotted for 3 s with filter paper before being plunged into liquid ethane and stored in liquid nitrogen. The quality of the grids was confirmed on a 200-kV Arctica electron microscope (Thermo Fisher Scientific) equipped with a K2 summit camera (Gatan). The large datasets were collected using SerialEM [36] on a Titan Krios electron microscope (Thermo Fisher Scientific) operated at 300 kV and at a nominal magnification of 105 000× using the objective lens defocus range of –1.0 to –2.0 μm. All EM images were recorded in the super-resolution counting and movie mode on a K3 summit camera (Gatan) with a dose rate of 0.85 electrons per Å^2^ per frame; a total of 60 frames were recorded in each movie micrograph. Sample preparation and data collection for the holoenzyme containing the dimer interface mutant polymerase were the same except that Quantifoil R1.2/1.3 Gold, 400-mesh grids were used, the dose rate was 0.92 electrons per Å^2^ per frame, and a total of 65 frames were recorded in each movie micrograph.

### Data processing

The polymerase complex dataset containing 12 045 movie stacks was processed with cryoSPARC (v4.2.1) [37]. The movies were drift-corrected with electron-dose weighting and two-fold binned using patch-based motion correction. In addition, the micrographs were CTF corrected with patch based contrast transfer function (CTF) estimation. The full dataset was split into three subsets with around 4000 movie stacks per subset. For each subset, the particles were auto-picked based on templates from the initial results and extracted with four-fold binning. After 2D classification, the “good” 2D class averages with defined structural features consisting of 922 085 particles were selected and merged as input for *ab initio* 3D model reconstruction resulting in a density map of polymerase complexes at 3.5 Å from 516 361 particles. This density map was used to train a Topaz [38] model to pick and extract 877 778 particles from the original uncorrected movie micrographs; the particles were downscaled by a factor of four for 2D classification. From this second 2D classification step, the “good” 2D class averages with defined structural features consisting of 738 880 particles were selected and merged as input for *ab initio* 3D model reconstruction. The 3D model reconstruction resulted in four density maps, only one of which included density corresponding to a two-polymerase complex (323 744 particles). This particle set was merged with the previous dataset of 516 361 particles. After removing the redundant particles, 647 230 particles underwent heterogeneous refinement to reveal two classes with the P/T DNA substrate associated with either the left or the right polymerase. An inspection of the density maps shows that the finger domain is not resolved in the class with the P/T DNA substrate associated with the left polymerase. Therefore, 3DVA was used to analyze the variation within this class revealing the presence of two subclasses depending on whether the DNA enters the polymerization active site of the left polymerase. All the density maps were further nonuniform refined to reach the final density maps of the three two-polymerase complexes identified as states 1, 2, and 3.

In the case of the dimer interface mutant gp43(exo-) polymerase complex, the data processing workflow from 10 316 movie stacks was similar. The initial template-based auto-picking, 2D classification, and *ab initio* 3D model reconstruction led to a density map from 417 703 particles. As before, this density map was used with Topaz model picking and selection of 2D classes to combine 340 304 particles with the first set of particles. After removing the redundant particles, the final dataset of 372 199 particles resulted in a 4.0 Å density map of the one-polymerase complex.

The resolution of all final maps was calculated based on the 0.143 threshold of the gold standard Fourier shell correlation between the two independently constructed “half” maps, with each “half” map using half of the dataset. The local resolution maps were calculated using the local resolution estimate program in cryoSPARC (v4.2.1) [37] and presented using UCSF ChimeraX [39].

### Modeling building and refinement

The initial atomic model for the T4 gp43 DNA polymerase was predicted with AlphaFold2; the domains of which were docked into the density maps and then the connecting loops were rebuilt in COOT [40]. The structure of the T4 gp45 sliding clamp, available in the PDB library, was docked into the density maps. The structure of the exact same P/T DNA substrate was previously solved with the yeast Pol δ [28] and docked into the density map as well and then adjusted in COOT [40]. Finally, the entire structural models were refined utilizing the PHENIX program [41] and validated using MolProbity [42, 43]. The T4 and RB69 DNA polymerase sequence alignment was generated using ESPript (https://espript.ibcp.fr) [44]. The structural model figures and alignments were generated using UCSF ChimeraX [39], and the sketches were created using Biorender.com. The structure and amino acid sequence of T4 exo- gp43 from this study were submitted to the ConSurf server [45] for residue conservation analysis. Default parameters were used, except that the sequence homologue identity range was set to 30%–90%.

## Results

### One sliding clamp binds one or two polymerases

Previous work has established that the T4 clamp loader is needed for loading and assembling T4 holoenzymes onto large linear or circular DNA substrates [7, 46], but is not necessary for *in vitro* assembly when the DNA substrate is short and the clamp has ready access to the DNA duplex end for direct binding [8]. Therefore, we assembled T4 polymerase–clamp complexes *in vitro* by directly combining individually purified gp43 polymerase and gp45 trimeric clamp proteins with a DNA substrate composed of a 25-nt primer annealed to a 38-nt template. The components were mixed at a molar ratio of 1.2 polymerase:2 clamp:1 DNA in the presence of 2′,3′-dideoxythymidine triphosphate (ddTTP), an incoming nucleotide analog (Fig. 1A). An exonuclease-deficient gp43 polymerase [gp43(exo-)], generated via the D219A mutation [35], was used to prevent the degradation of the DNA substrate during complex assembly.

Two-dimensional classification of cryo-EM particle images collected from the vitrified reaction mixture revealed two distinct complexes: a minor species containing a single polymerase bound to one sliding clamp and DNA substrate (one-polymerase complex), and a predominant species featuring two polymerases bound to the same sliding clamp and substrate (two-polymerase complex) (Fig. 1B and Supplementary Figs 1 and 2). The one-polymerase complex represented only ∼10% of the particle population and exhibited preferred orientation, resulting in a low-resolution 3D reconstruction (Supplementary Fig. 1). Owing to the limited resolution, we do not discuss the one-polymerase complex further here, but revisit it below in the context of a dimer-interface mutant polymerase and a functional T4 holoenzyme structure containing a single polymerase.

The two-polymerase complex accounted for ∼90% of the particle population, with polymerase dimerization likely resulting from the high sample concentration employed in cryo-EM studies (see Methods and materials). Three-dimensional classification of these two-polymerase complex particles yielded three distinct 3D EM maps, designated as states 1, 2, and 3, with overall resolutions of 3.57 Å, 3.7 Å, and 3.74 Å, respectively (Supplementary Table 1, Fig. 1B–D, and Supplementary Figs 1–3). Atomic models were constructed for each EM map through manual building of residues in higher-resolution regions and integration of AlphaFold models in lower-resolution mobile domains (Fig. 1E and F). All three states exhibit similar dimensions of ∼115 Å in height and 140 Å in width, sharing a common architecture featuring a single clamp ring that coordinates two face-to-face polymerases. And gp45 remains rigid throughout the process at the resolution achieved in our structures. Alignment of gp45 from the three states yields an average Cα RMSD of less than 1 Å, indicating that the clamps are nearly identical and undergo no detectable conformational changes. The three structures of the two-polymerase complex likely capture three intermediate states of polymerase exchange, as will be discussed in greater detail below.

### DNA path varies in the three two-polymerase complexes

The three two-polymerase states exhibit distinct DNA paths. In side-view projections of the 3D EM maps with the sliding clamp positioned at the top, the two polymerases flank the left and right sides of the clamp’s three-fold symmetry axis (Fig. 1B–D). All six polymerases display clear resolution across the three states (Fig. 1A and E). Notably, particle distribution shows nearly equivalent populations with bound DNA entering the right polymerase (state 1; 54%) compared to those with DNA entering the left polymerase (states 2 and 3 combined; 46%). In both states 1 and 2, the DNA substrate remains displaced from the polymerization active site in either polymerase, with the primer 3′ terminus positioned 16 Å away from the catalytic residue Asp-620 and the finger domain maintaining an open conformation. Conversely, in state 3, representing 20.6% of the particle population, the primer 3′ terminus successfully engages the polymerization active site of the left polymerase, triggering finger domain closure that stabilizes the incoming ddTTP nucleotide analogue (Supplementary Fig. 4).

### Asymmetric polymerase arrangement with an extensive interface

**Figure 2.**
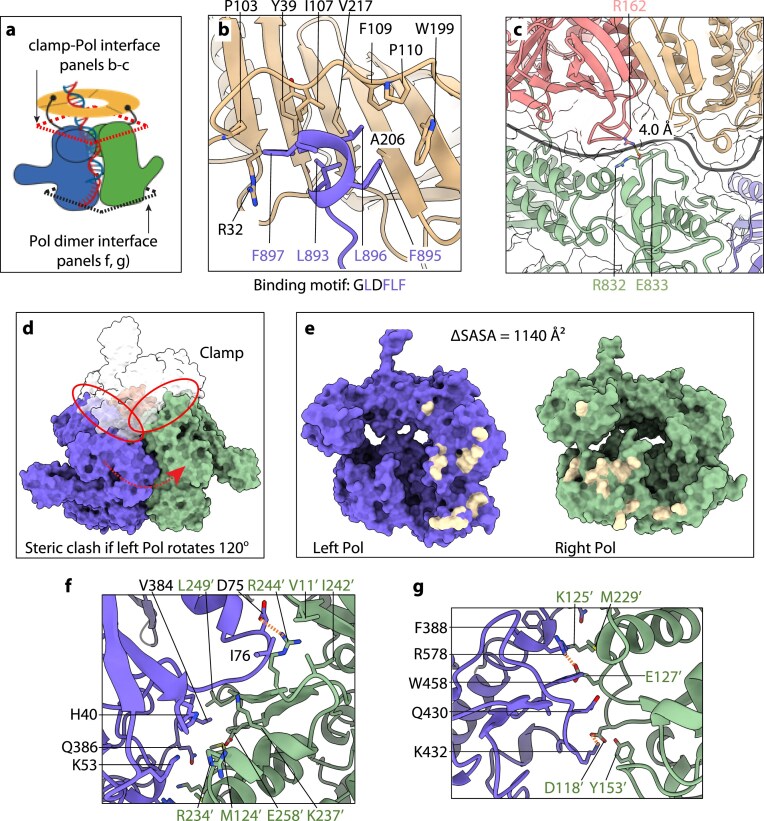
Protein-protein interfaces in the T4 two-polymerase exchange complex, illustrated using state 3 as an example. (**A**) Schematic of the two-polymerase complex showing the two gp43(exo-) bound to the gp45 clamp (yellow) through their CIP (black curves ending in dots). The dashed red rhombus marks the interface between the clamp and polymerase, explored in panels (B) and (C), while the dashed black rhombus denotes the primary dimer interface between the polymerases, detailed in panels (F) and (G). Created in BioRender (https://BioRender.com/bfkkkot). (**B**) The CIP of the blue polymerase inserts into a hydrophobic pocket on the clamp (tan). (**C**) Residue R162 of the clamp forms a weak H-bond (4 Å) with residue E833 of the right polymerase. The thick black curve delineates the boundary between the clamp and the polymerases. (**D**) The left and right polymerases are not symmetric equivalents. A 120° rotation of the left polymerase around the clamp three-fold axis results in steric clashes with the clamp (red ovals), preventing superposition with the right polymerase. (**E**) The two polymerases are in an open-book view to reveal the dimer interface, with interacting residues highlighted in light yellow. (**F, G**) Detailed views of the inter-polymerase contacts in the upper (F) and lower (G) regions of the dimer interface.

Within each T4 gp45 clamp monomer, the linker loop and gap separating the NTD and CTD form a hydrophobic pocket that accommodates the polymerase C-terminal CIP sequence (892-SLDFLF-897) (Fig. 1A and F). The T4 polymerase CIP adopts a single-turn α-helical conformation (Fig. 2A and B), extending slightly beyond the canonical 3^10^-helix motif [14]. We note that the CIPs of both gp43 polymerases engage the clamp simultaneously, and these interactions are maintained across all three states observed. Additional clamp-polymerase contacts can involve the polymerase thumb domain of the non-DNA interacting polymerase, which engages the clamp through a salt bridge linking clamp residue Arg-162 with polymerase residue Glu-833 in state 3 (Fig. 2C). Although this interaction is weak (4.0 Å), the interacting regions exhibit complementary geometry.

Inside the clamp channel, an extended loop/turn region (M184-N193) substitutes for one of the four α-helices (H4) typically found in cellular sliding clamps (H1/H2 in NTD and H3/H4 in CTD) [22, 23] (Fig. 1F). This structural modification transforms the central channel from the circular geometry characteristic of cellular clamps to a triangular configuration [47] (Supplementary Fig. 5). The substantially larger polymerase size relative to the gp45 clamp subunit restricts binding to only two polymerases per clamp ring, as insufficient space exists to accommodate a third polymerase. This size disparity also necessitates asymmetric polymerase positioning, since symmetric arrangement through 120° rotational relationships around the clamp’s three-fold axis would generate steric conflicts between the polymerases (Fig. 2D).

The polymerase dimer interface spans 1140 Å^2^ and features complementary surface topography and electrostatic properties between the left polymerase NTD/palm domain and the right polymerase NTD/exonuclease domain (Fig. 2E). The interface’s upper region involves hydrophobic contacts between Val-384 and Ile-76 from the left polymerase with Leu-249, Val-11, and Ile-242 from the right polymerase, while Asp-75 of the left polymerase establishes a salt bridge with Arg-244 of the right polymerase (Fig. 2F). The central and lower interface sections feature multiple polar interactions, including two prominent salt bridges: one between Arg-578 of the left polymerase with Glu-127 of the right polymerase, and another between Lys-432 of the left polymerase with Asp-118 of the right polymerase (Fig. 2G).

### DNA-polymerase interactions within the two-polymerase complex

**Figure 3.**
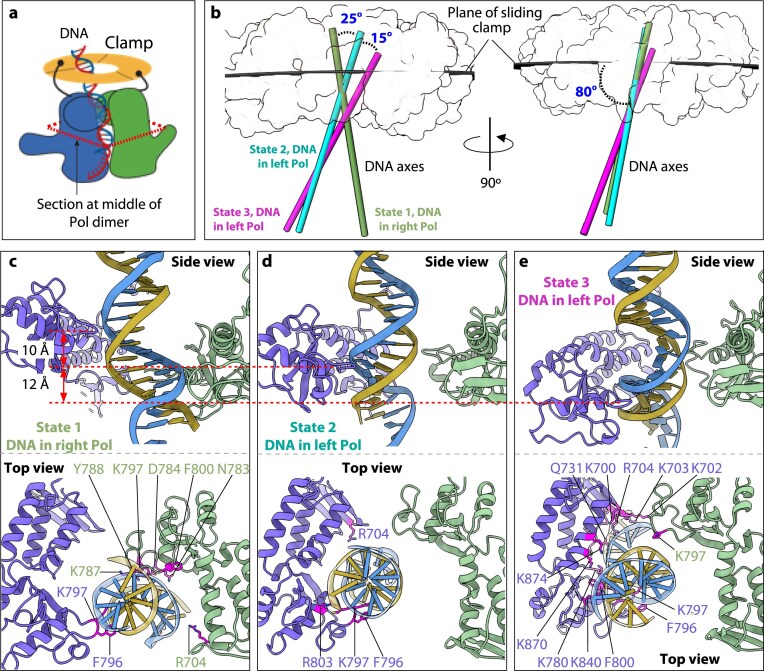
Progressive repositioning of DNA during transitions between states 1–3 of the two-polymerase complex. (**A**) Diagram of the T4 two-polymerase exchange complex with two gp43(exo-) polymerases (blue and green) bound to the gp45 clamp (yellow) via the CIP (black curves ending in dots). The dashed red rhombus marks the DNA interaction region. Created in BioRender (https://BioRender.com/bfkkkot). (**B**) Orthogonal views showing the orientations of the DNA duplex in state 1 (dark green), state 2 (cyan), and state 3 (magenta). The clamp is depicted as a light gray surface; polymerases and P/T DNA are omitted for clarity. (**C–E**) Side and top views of the DNA substrate at the polymerase dimer interface. The DNA is directed towards the right polymerase in state 1 (C), shifts towards the left polymerase but does not reach the polymerization site in state 2 (D), and occupies the active site of the left polymerase in state 3 (E). The dashed red lines indicate the downward displacement of the left polymerase helical domain (blue), which moves ∼10 Å between states 1 and 2, and an additional 12 Å between states 2 and 3 to guide the DNA into the active site.

Structural alignment of the sliding clamps demonstrates that the P/T DNA across all three states deviates significantly from perpendicular orientation relative to the clamp plane, displaying pronounced tilting away from the clamp’s three-fold symmetry axis (Fig. 3A and B). The polymerase thumb domains dynamically track the evolving DNA duplex trajectory throughout these conformational states (Fig. 3C–E). In state 1, the left polymerase thumb is elevated 10 Å higher than its right counterpart (Fig. 3C) while the left polymerase thumb residues Lys-797 and Phe-796 bind the duplex DNA. Concurrently, the right polymerase thumb establishes DNA contacts through residues Asn-783, Asp-784, Lys-787, Tyr-788, Lys-797, and Phe-800, which engage the DNA minor groove, while Arg-704 contacts the DNA backbone. The primer 3′-terminus remains excluded from both polymerization and exonuclease active sites of the right polymerase. In state 2, both thumb domains align at equivalent heights (Fig. 3D). DNA interaction is restricted to the left polymerase, where thumb residues Arg-704, Phe-796, Lys-797, and Arg-803 engage the substrate, while the right polymerase maintains no DNA contacts. The primer 3′-end occupies neither catalytic site of the left polymerase. State 3 exhibits a dramatic 12 Å downward shift of the left polymerase thumb domain, establishing extensive DNA interactions through eight positively charged residues (Lys-840, Lys-780, Lys-870, Lys-874, Lys-700, Arg-704, Lys-703, and Lys-702) that direct the primer 3′-terminus into its polymerization active site (Fig. 3E). Thus, the left polymerase in state 3 adopts a catalytically competent configuration.

### Conformational changes transitioning DNA into a catalytically competent position

**Figure 4.**
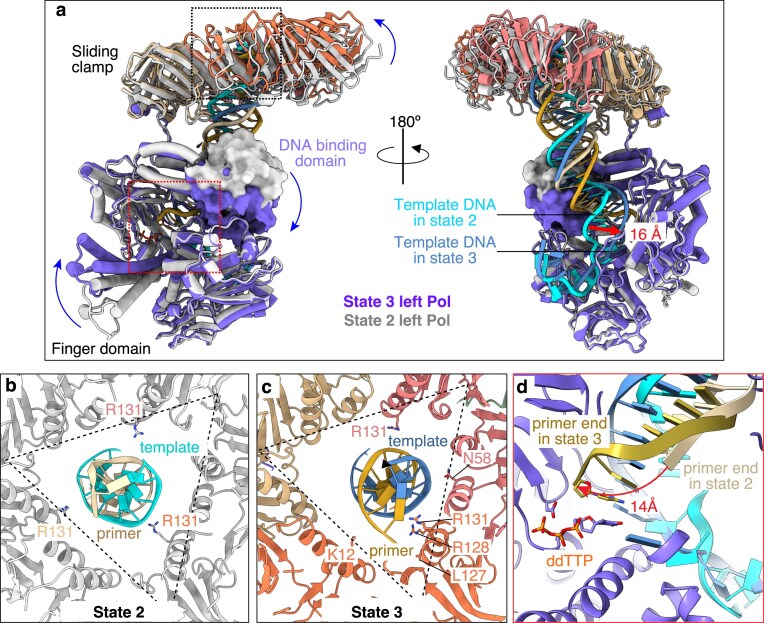
Structural rearrangement of the left polymerase during the transition from state 2 to state 3 of the two-polymerase complex. (**A**) The DNA-bound left polymerases of state 2 (gray) and state 3 (purple) are superimposed and displayed in front and back orientations. The right polymerases are omitted for clarity. Curved blue arrows denote the relative movements of the polymerase domains and the clamp. (**B, C**) Detailed views of DNA engagement within the interior channel of the clamp for state 2 (**B**) and state 3 (**C**). Dashed triangles outline the clamp’s overall channel geometry. (**D**) Overlay of the DNA substrates bound to the left polymerase in state 2 (light brown primer, cyan template) and state 3 (gold primer, blue template). The primer 3′-terminus undergoes a ∼14 Å shift to reach the polymerization active site in state 3. The incoming nucleotide analog dTTP in state 3 is depicted as sticks and labeled.

Across states 1–3, the right polymerase maintains structural consistency while the left polymerase undergoes significant conformational changes. Superposition of the right polymerases of states 2 and 3 illuminates the structural rearrangements required to reposition the DNA primer from its inactive placement in the left polymerase of state 2 to the catalytically competent configuration observed in state 3 (Fig. 4A–D). This alignment reveals four coordinated transitions: (i) further upward tilting of the sliding clamp relative to the DNA; (ii) downward compression of the polymerase thumb for improved DNA engagement; consequently, (iii) P/T DNA rotation that shifts the template strand laterally by 16 Å to optimize interactions with the thumb; and (iv) finger domain closure to stabilize the incoming nucleotide (Fig. 4A).

In state 2, the DNA occupies the central region of the clamp channel, maintaining proximity to all three Arg-131 residues from each clamp monomer (Fig. 4B). However, state 3 displays increased DNA tilting that directs the substrate toward the lower vertex of the triangular clamp channel, facilitating interactions with Arg-128 and Leu-127 within a single clamp monomer (Fig. 4C). Comparative analysis of the left polymerase conformations between states 2 and 3 demonstrates a 14 Å translocation of the primer 3′-terminus to occupy the polymerization active site (Fig. 4D). Additionally, the closed finger domain stabilizes base stacking between the incoming ddTTP nucleotide analog and the primer terminal base, establishing the catalytically competent configuration [48, 49].

We note that the extended template strand executes a nearly 180° turn, occupying the anticipated template entry pathway for the opposing polymerase within the dimer (Fig. 4A, right panel). This template strand configuration represents an *in vitro* artifact due to the experimental DNA substrate design, which incorporates a 13-nt 5′-template overhang exceeding the length required for polymerase binding. In a functioning T4 replisome, the incoming template would normally derive from parental double-stranded DNA in leading-strand holoenzyme contexts or would be coated with gp32 ssDNA-binding protein in lagging-strand holoenzyme contexts.

### Structure of the one-polymerase T4 holoenzyme

We used ConSurf [45] to analyze 28 phage polymerase homologues spanning 30%–90% sequence identity and found that the dimer interface is not well conserved (Supplementary Fig. 6). This indicates that polymerase dimerization on the clamp is not a universal feature in phages.

**Figure 5.**
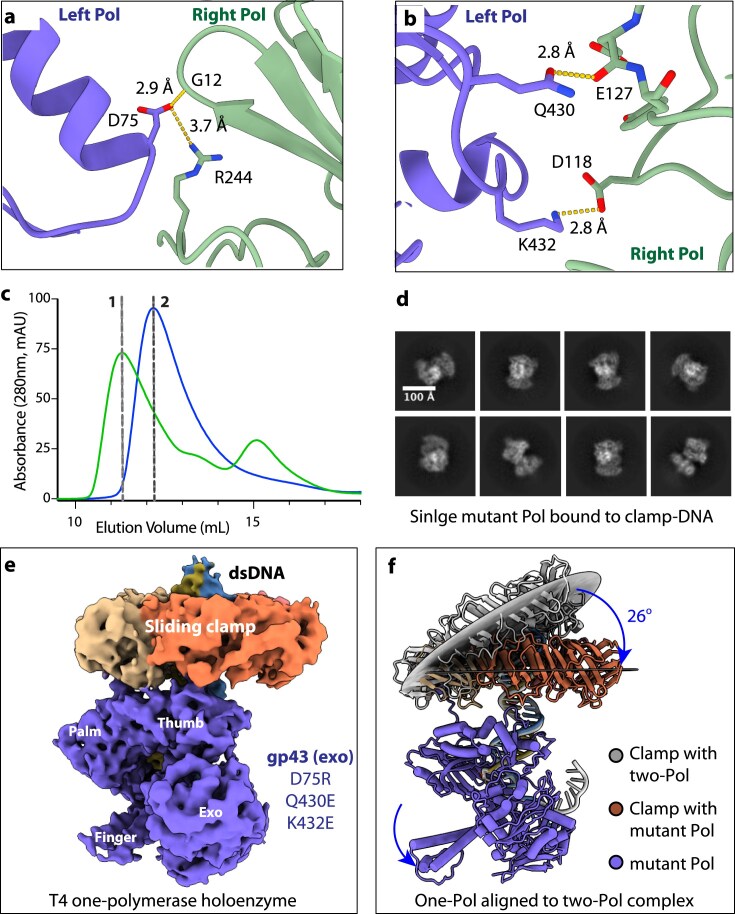
Mutations disrupting the polymerase dimer interface yield the one-polymerase T4 holoenzyme. (**A, B**) The dimer interface in the two-polymerase complex (state 3) is stabilized by three salt bridges (shown as sticks). Mutations D75R, Q430E, and K432E were introduced into gp43(exo-) to disrupt this interface. (**C**) Size-exclusion chromatography of mixtures containing the gp45 clamp and P/T DNA with either gp43(exo-) (green) or the dimer-interface mutant (blue) polymerase demonstrates the loss of the dimer formation and assembly of a one-polymerase holoenzyme. Dashed gray lines denote the expected elution positions for complexes containing two polymerases (line 1) or one polymerase (line 2). (**D**) Representative 2D class averages of the holoenzyme formed with the mutant polymerase. (**E, F**) EM map (E) and structural model (F) of the one-polymerase T4 holoenzyme, showing a 26° downward tilt of the clamp relative to the two-polymerase complex in state 3.

To obtain the structure of a one-polymerase complex that more closely aligns with the configuration observed in other B-family polymerase holoenzymes, we strategically disrupted key dimer interface residues. Three mutations (D75R, Q430E, and K432E) were incorporated into the gp43(exo-) polymerase to break two critical salt bridges (Asp75:Arg244 and Lys432:Asp118) and eliminate one hydrogen bond (Gln430:Glu127) within the dimer interface (Fig. 5A and B). Following successful expression and purification of this dimer-interface mutant (Supplementary Fig. 7), size exclusion chromatography demonstrated that the complexes assembled with the dimer-interface mutant polymerase exhibited delayed elution compared to those containing the gp43(exo-) polymerase (Fig. 5C). This elution shift indicated effective disruption of the polymerase dimer interface, yielding T4 holoenzyme complexes with only one polymerase. Cryo-EM analysis provided additional confirmation, with 2D class averages clearly showing the presence of only one polymerase (Fig. 5D). In principle, two flexible polymerases could each engage the same clamp via their respective CIP motifs. Nevertheless, the absence of detectable binding of two dimer-interface mutant gp43 molecules to one gp45 clamp indicates that electrostatic repulsion between the mutants destabilizes this configuration, rendering it energetically unfavorable.

We subsequently determined the cryo-EM structure of this one-polymerase T4 holoenzyme at an overall resolution of 4.0 Å (Fig. 5E and Supplementary Figs 8 and 9). A striking feature of this structure is the altered orientation of the sliding clamp, which tilts downward 26° relative to its position in the two-polymerase complex, positioning it perpendicular to the DNA (Fig. 5F). The primer 3′-terminus occupies the polymerization active site, suggesting an active conformation, although the finger domain remains open in anticipation of an incoming nucleotide binding (Supplementary Fig. 4E). This structure thus defines the canonical T4 polymerase holoenzyme architecture, in which a single polymerase associates with the sliding clamp that adopts a largely perpendicular orientation relative to the DNA.

### Active T4 holoenzyme consists of one polymerase and clamp

**Figure 6.**
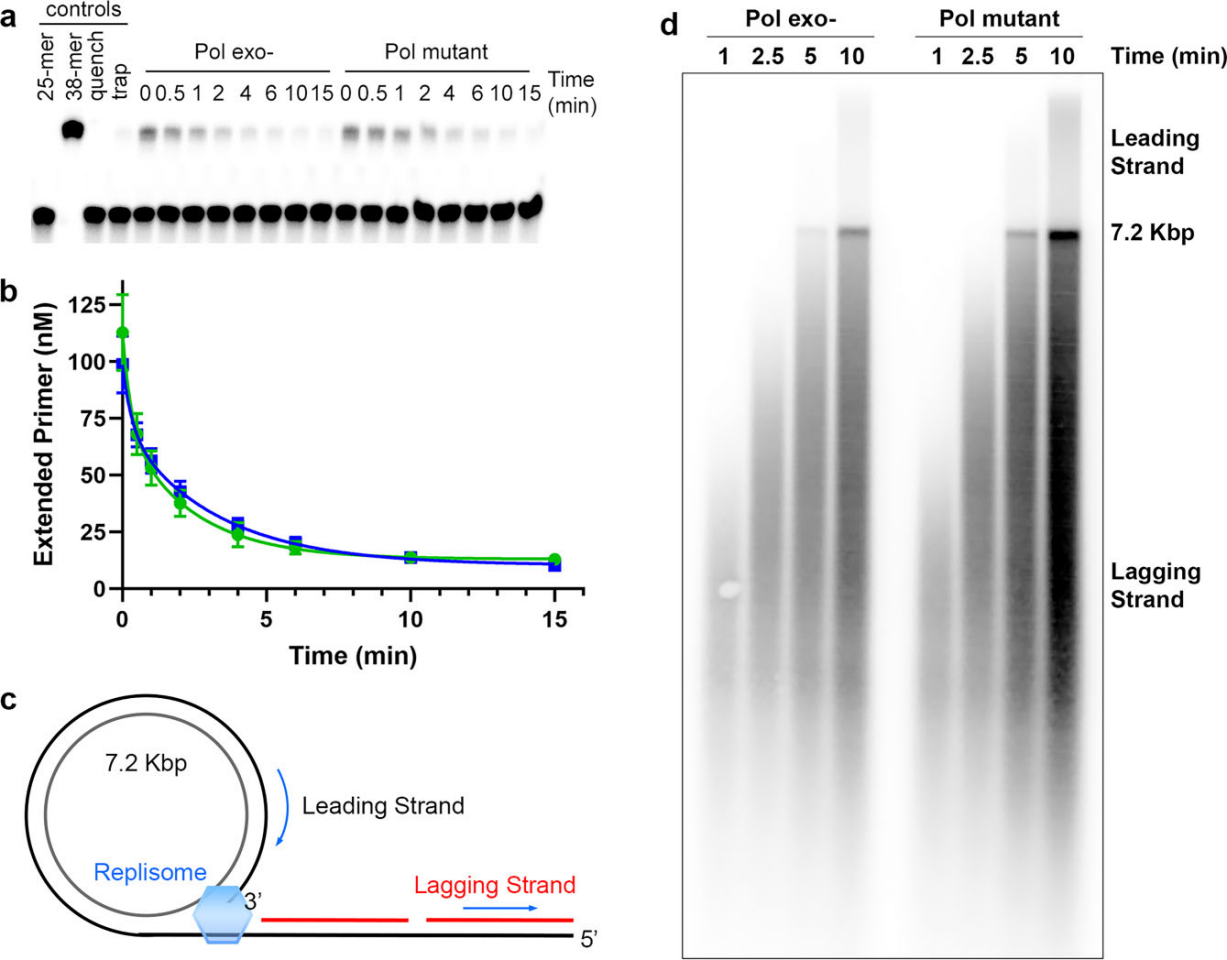
Functionality of holoenzymes with gp43(exo-) and dimer-interface mutant polymerases. (**A**) One-polymerase holoenzyme complexes were formed using either gp43(exo-) or dimer-interface mutant polymerases on a blocked P/T DNA substrate. Following the addition of a single-stranded DNA trap, the holoenzymes were allowed to decay for defined time periods before the introduction of deoxynucleotides to assess the fraction of holoenzyme that remained bound to the P/T DNA. Holoenzyme retention on P/T DNA was determined at the indicated time point by denaturing PAGE, revealed as the extended 38-mer product. (**B**) Quantification at *t* = 0 min confirmed that nearly stoichiometric holoenzyme complexes (one polymerase per clamp) were formed using either polymerase at 100 nM concentration. Holoenzymes containing gp43(exo-) (green circles) and dimer interface mutant polymerases (blue squares) displayed comparable stability and dissociation kinetics, with no detectable differences between the two variants. (**C**) Schematic illustration of replisome positioning on a 7.2-kbp DNA substrate and the resulting products generated in rolling circle assays. d) Denaturing alkaline agarose gel analysis of DNA synthesized by gp43(exo-) and dimer-interface mutant polymerases shows comparable activity and processivity on both leading and lagging DNA strands.

We examined the functional properties of holoenzyme complexes assembled with either gp43(exo-) or the dimer-interface mutant polymerase. A modified P/T DNA substrate was utilized, featuring biotin and neutravidin blocks at both ends of the template strand to prevent polymerase–clamp complexes from diffusing off the small DNA substrate. These terminal blocks necessitated the use of gp44/62 clamp loader for positioning clamp on DNA and subsequent polymerase recruitment to form holoenzyme complexes. Complex stoichiometry and stability were assessed by introducing an ssDNA trap to block new holoenzyme assembly, followed by deoxynucleotide additions at various time points. Reaction conditions included a five-fold molar excess of P/T DNA (0.5 µM), clamp, and clamp loader over polymerase (0.1 µM), establishing polymerase as the limiting component for holoenzyme assembly. Both gp43(exo-) and dimer-interface mutant polymerases initially formed complexes on ∼20% (0.1 µM) of available P/T DNA substrates at the zero-time point (Fig. 6A and B), indicating that each polymerase variant produced 0.1 µM holoenzymes with a 1:1 clamp-to-polymerase ratio. The predominant two-polymerase complexes observed in cryo-EM studies were assembled using 6 μM gp43(exo-). The 60-fold reduction in polymerase concentration employed in these functional assays (0.1 μM) most likely explains the exclusive formation of one-polymerase complexes, although we cannot exclude potential effects from the clamp loader, which was omitted from cryo-EM sample preparations. Time-course analysis of holoenzyme retention on the P/T DNA substrate demonstrated that gp43(exo-) polymerase stability or dissociation kinetics (k_off_ ≈ 0.007 s^−1^) closely resembles that of the dimer-interface mutant polymerase (k_off_ ≈ 0.005 s^−1^) (Fig. 6A and B). These dissociation constants align with previously reported measurements [7, 46], indicating that holoenzyme stability depends primarily on clamp subunit interactions and the influence of biotin/neutravidin terminal blocks on short DNA substrates under these *in vitro* conditions.

A rolling circle assay was additionally performed to compare the *in vitro* performance of gp43(exo-) and dimer-interface mutant polymerases within fully reconstituted T4 replisomes assembled on tailed circular 7.2 kb DNA substrates, permitting concurrent leading- and lagging-strand synthesis (Fig. 6C). Over a 10-min time course, the dimer-interface mutant polymerase, which is incapable of forming dimeric polymerase complexes, displayed equivalent or slightly superior activity and processivity for both replication strands compared to the gp43(exo-) polymerase, which maintains the potential to dimerize within clamp-containing complexes (Fig. 6D). Collectively, these results support our conclusion that the two-polymerase complexes detected at elevated protein concentrations do not constitute the active DNA-synthesizing holoenzyme, but instead represent transient intermediates generated during the polymerase exchange process.

## Discussion

**Figure 7.**
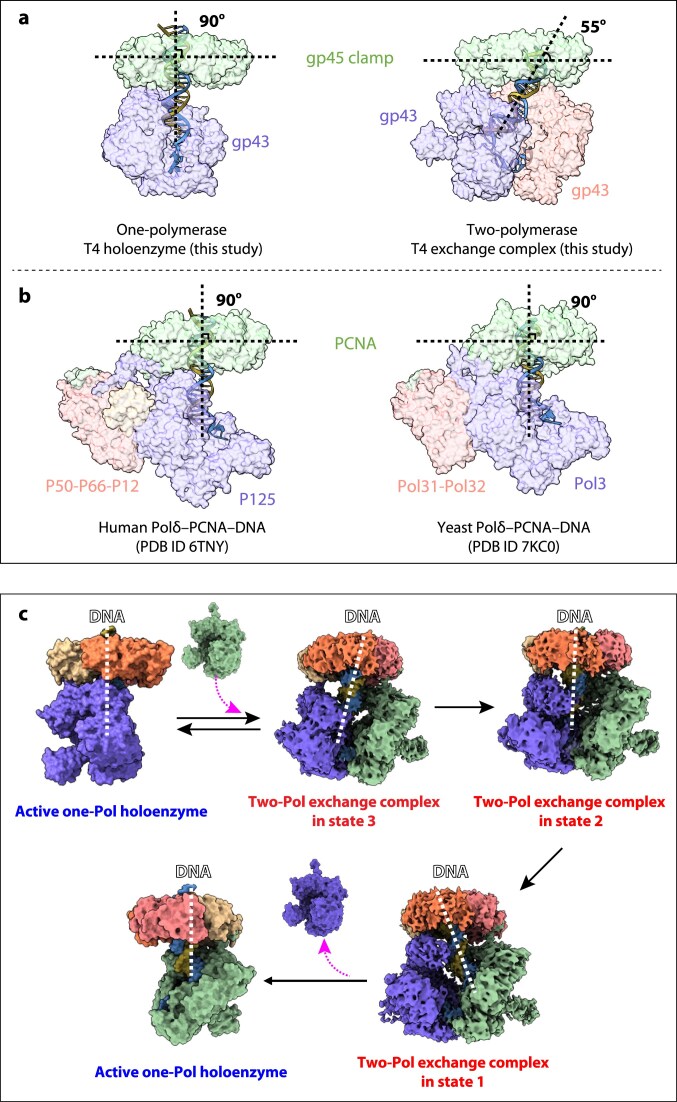
Structural comparison of replicative holoenzymes and model for T4 polymerase exchange. (**A**) In the T4 holoenzyme assembled with the dimer-interface mutant polymerase (left), the DNA duplex is positioned nearly perpendicular to the clamp plane, whereas in the two-polymerase exchange complex (right), the DNA displays a ∼55° tilt. (**B**) Comparison of human (left) and yeast (right) Polδ holoenzyme structures demonstrates that cellular replicative holoenzymes generally maintain DNA in a perpendicular orientation relative to the sliding clamp. (**C**) Schematic of the proposed T4 polymerase exchange mechanism, in which the indicated steps may occur reversibly.

DNA replication in T4 bacteriophage is carried out by a primary holoenzyme complex that most likely features a single DNA polymerase bound to the sliding clamp that encircles the nascent DNA. Cryo-EM analysis of a dimer-interface mutant polymerase has revealed the structural organization of this one-polymerase complex. The resulting structure shows the clamp positioned perpendicular to the DNA, consistent with other B-family holoenzyme structures [27, 28] (Fig. 7A and B). This perpendicular arrangement allows the sliding clamp to move along the DNA on a thin film of water molecules known as the “water skating” mechanism [29].

Cryo-EM studies conducted at elevated polymerase concentrations successfully captured three distinct two-polymerase complexes. Alberts and coworkers initially hypothesized the existence of a polymerase dimer in the T4 replisome, suggesting it could facilitate rapid recycling of the lagging-strand polymerase following Okazaki fragment completion [31]. Compelling experimental support for the coupling of leading- and lagging-strand holoenzyme complexes derives from the observation that DNA synthesis on both strands remains unaffected when reaction components are diluted [32]. Evidence for the physical association of two gp43 polymerases includes the retention of gp43 polymerase from clarified T4-infected *E. coli* lysates using gp43 polymerase affinity chromatography [31], as well as results from an *in vivo* two-hybrid assay employing gp43–λ cI repressor fusion proteins in *E. coli* to identify specific gp43 polymerase domains responsible for homodimer assembly [50]. Nevertheless, the two-polymerase complexes we observed do not constitute the leading- and lagging-strand polymerase dimer found in a T4 replisome, since our cryo-EM complexes contain only one clamp. Dilution experiments have demonstrated that each lagging-strand polymerase recycling event for new Okazaki fragment initiation requires loading a fresh clamp from solution onto the DNA [32, 51, 52]. Therefore, the polymerase dimer interface we characterized differs from that proposed by Alberts that coordinates leading- and lagging-strand replication in a T4 replisome.

We propose that the three structurally distinct two-polymerase complexes identified by cryo-EM represent sequential intermediates in the polymerase exchange pathway rather than nonproductive or aberrant structures. The cryo-EM experiments employed polymerase concentrations 10-fold above physiological levels, and under these conditions, 90% of particles contained two polymerases while 10% contained only one polymerase. This distribution suggests that two-polymerase complex formation is concentration-dependent, a conclusion reinforced by observations that polymerase exchange itself exhibits concentration dependence even at much lower polymerase levels, as discussed below. Additional evidence for these being functional intermediates comes from the structural differences between the two-polymerase complexes: state 3 (comprising ∼21% of two-polymerase complexes) shows primer/template DNA in an active, engaged conformation at the polymerization site, whereas states 1 and 2 position the primer/template outside this site. Importantly, if rapid interconversion among the two-polymerase states and with the one-polymerase complex did not occur, accumulation of the inactive states 1 and 2 would slow polymerization kinetics at elevated polymerase concentrations—an effect that has not been observed experimentally. Collectively, these findings support the model that these complexes are functional intermediates in the exchange mechanism rather than off-pathway products.

The phenomenon of polymerase exchange, where solution-phase polymerase proteins replace those actively synthesizing DNA within a replisome, has been observed in the T4 replication system. Previous experiments employed a catalytically inactive T4 gp43 polymerase “trap” containing D219A and D408N mutations that eliminate exonuclease and polymerization activity, respectively, while preserving wild-type binding properties [33]. When this trap polymerase was introduced to active replisomes operating on rolling circle DNA substrates, both leading- and lagging-strand polymerases showed identical inhibition levels within 1 min. The polymerase exchange followed an active displacement mechanism, proceeding much more rapidly than the measured dissociation rate of T4 holoenzymes and showing clear dependence on polymerase concentration—higher polymerase concentrations led to more frequent exchanges. Even at the relatively low trap concentration of 100 nM, the polymerase exchange rate reached ∼0.02 s⁻¹, which is 10-fold higher than the normal polymerase dissociation rate. Since the trap polymerase retains wild-type binding characteristics, this indicates that similar exchanges occur among wild-type polymerases. Based on the estimated *in vivo* gp43 concentration of ∼600 nM [53], calculations suggest that polymerase exchange occurs roughly every 10 s within any given replisome. This translates to ∼90 exchange events per replication fork during the 15-min period required to complete T4 genome replication [33]. While the actual duration of each exchange event remains unmeasured, the process appears to be rapid since no detectable pauses in DNA synthesis rates have been observed.

The molecular mechanism of replicative polymerase exchange is revealed through three distinct two-polymerase complexes captured by cryo-EM (Fig. 7C). Across the three states, the DNA is severely tilted against the sliding clamp (Fig. 7A, right panel). This tilted configuration prevents the complexes from sliding along DNA effectively stalling the holoenzyme and DNA replication [29]. The exchange process begins when a second polymerase binds to an active one-polymerase holoenzyme through additional CIP binding sites on the clamp, positioning itself to the right of the active polymerase via dimer interface interactions. The incoming polymerase pushes the sliding clamp upward by 26°, creating a tilted DNA orientation that may prevent the polymerase–clamp complex from sliding away from the P/T junction [29]. The primer 3′-terminus remains in the polymerization active site of the original polymerase in this initial intermediate represented by state 3. During the transition from state 3 to state 2, the duplex region of the DNA moves toward the clamp’s central channel and becomes less severely tilted. This shift causes the primer/template DNA to disengage from the left polymerase and move 14 Å toward the right polymerase. As the polymerase exchange progresses from state 2 to state 1, the duplex DNA tilts by 25° toward the right polymerase allowing the P/T junction to approach the polymerization active site of the right polymerase. The sliding clamp adjusts its position to accommodate this DNA movement, and this repositioning causes the original left polymerase to dissociate from the clamp. Finally, once the left polymerase departs, the right polymerase undergoes additional conformational changes while the clamp tilts downward becoming perpendicular relative to the DNA. This completes the formation of a new active one-polymerase holoenzyme with the replacement polymerase now in control of DNA synthesis.

Other bacteriophage and bacterial reconstituted replication systems exhibit highly dynamic polymerase exchange. The bacteriophage T7 replication system exemplifies dynamic polymerase processivity through multiple interaction modes with the helicase protein [54, 55]. T7 DNA polymerase binds the helicase tightly when engaged with primer-template DNA, but interacts more weakly through electrostatic contacts between basic loops in the polymerase C-terminus and the acidic C-terminal tail of helicase when DNA-free [56, 57]. This dual binding mode allows the polymerase to transiently dissociate from DNA while remaining tethered to the replication fork via helicase interactions. The helicase can bind “spare” polymerases in this weaker mode, enabling their exchange onto the template during dissociation events of the active polymerase. Single-molecule studies have confirmed the presence of two or more polymerases in the T7 replisome during replication [58, 59]. Structural analysis revealed that two DNA polymerase molecules bind the ring-shaped primase-helicase in a conserved orientation facilitating polymerase loading onto DNA, while a third polymerase binds in an offset manner that may enable exchange [60]. Thus, the helicase may travel with multiple polymerases—two engaged and one in reserve—periodically switching to the spare to maintain high processivity while facilitating polymerase exchange.

The bacterial replication system is more complex than bacteriophage systems, featuring multiple polymerase exchange pathways. Similar to T7, *E. coli* may maintain a backup polymerase in its replisome. In *E. coli*, the clamp loader is a pentameric complex containing three DnaX subunits (γ or τ) plus δ and δ' subunits. Complexity stems from the dnaX gene producing both τ and γ proteins at equal ratios through programmed translational frameshifting, with γ being a truncated version. DNA polymerase III core binds tightly to τ’s unique C-terminal domains. The clamp loader composition has been debated. Initial evidence suggested a τ₃δδ' complex could load three polymerase III cores [61, 62], but later studies indicate the natural clamp loader composition may be γτ₂δδ', accommodating only two polymerase III cores [63]. Beyond the replicative polymerase and clamp loader, β clamps interact with numerous proteins. These include DNA polymerase I and ligase I for Okazaki fragment processing [64], and translesion polymerases (Pol II, IV, and V) that bypass DNA lesions blocking Pol III (reviewed in [65]). These proteins compete for the same clamp binding site, a feature shared with eukaryotic PCNA [66]. The clamp can simultaneously bind multiple factors, functioning as a molecular “toolbelt” carrying repair proteins during replication [67, 68]. For example, β clamp can form a ternary complex with Pol IV and Pol III, enabling rapid lesion bypass by Pol IV followed by immediate replacement with high-fidelity Pol III.

Furthermore, studies have shown that the eukaryotic replicative polymerases, especially Polα and Polδ, undergo relatively rapid exchange events during *in vitro* DNA synthesis [69, 70]. Thus, polymerase exchange occurs across all replication systems, from simple bacteriophage replicases to sophisticated eukaryotic replisomes, indicating this is an evolutionarily conserved mechanism crucial for maintaining genome stability. This process enables error correction, navigation of DNA template obstacles, and replacement of defective polymerases while preserving replisome integrity—essential for efficient and timely genome duplication. Our cryo-EM structural analysis of the T4 holoenzyme captures three discrete conformational states within a two-polymerase/clamp complex, revealing a multi-step exchange mechanism. These structures provide the first atomic-resolution view of replicative polymerase switching and establish a structural framework for understanding how replisomes dynamically coordinate multiple polymerases to ensure faithful DNA replication. This mechanism likely underlies polymerase coordination in more complex systems, suggesting new avenues for investigating replication fidelity and repair in higher organisms.

## Supplementary Material

gkaf1359_Supplemental_File

## Data Availability

Cryo-EM 3D maps and atomic models of the T4 two-polymerase complex in states 1, 2, and 3 have been deposited in the Electron Microscopy Data Bank (EMDB) and Protein Data Bank (PDB) under accession codes EMDB-47826/PDB 9EA6, EMDB-47531/PDB 9E5Y, and EMDB-47824/PDB 9EA2, respectively. The 3D map and atomic model of the dimer-interface mutant one-polymerase T4 holoenzyme have been deposited under the accession codes EMDB-47825 and PDB 9EA3, respectively.

## Notes

Present address: Biology and Biological Engineering, Caltech, 1200 E. California Blvd., Pasadena, CA 91125, United States

## References

[1] McCarthy D, Minner C, Bernstein H et al. DNA elongation rates and growing point distributions of wild-type phage T4 and a DNA-delay amber mutant. J Mol Biol. 1976;106:963–81. 10.1016/0022-2836(76)90346-6. 789903

[2] Fangman WL, Brewer BJ. A question of time: replication origins of eukaryotic chromosomes. Cell. 1992;71:363–6. 10.1016/0092-8674(92)90505-7. 1423601

[3] Alberts B, Johnson A, Lewis J et al. Molecular Biology of the Cell. 4th edition. New York: Garland Science, 2002.

[4] Drake JW . A constant rate of spontaneous mutation in DNA-based microbes. Proc Natl Acad Sci USA. 1991;88:7160–4. 1831267 10.1073/pnas.88.16.7160 PMC52253

[5] Santos ME, Drake JW. Rates of spontaneous mutation in bacteriophage T4 are independent of host fidelity determinants. Genetics. 1994;138:553–64. 10.1093/genetics/138.3.553. 7851754 PMC1206207

[6] Kaboord BF, Benkovic SJ. Rapid assembly of the bacteriophage T4 core replication complex on a linear primer/template construct. Proc Natl Acad Sci USA. 1993;90:10881–5. 10.1073/pnas.90.22.10881. 8248185 PMC47882

[7] Kaboord BF, Benkovic SJ. Dual role of the 44/62 protein as a matchmaker protein and DNA polymerase chaperone during assembly of the bacteriophage T4 holoenzyme complex. Biochemistry. 1996;35:1084–92. 10.1021/bi9520747. 8547244

[8] Reddy MK, Weitzel SE, von Hippel PH. Assembly of a functional replication complex without ATP hydrolysis: a direct interaction of bacteriophage T4 gp45 with T4 DNA polymerase. Proc Natl Acad Sci USA. 1993;90:3211–5. 8475061 10.1073/pnas.90.8.3211 PMC46269

[9] Jarvis TC, Newport JW, von Hippel PH. Stimulation of the processivity of the DNA polymerase of bacteriophage T4 by the polymerase accessory proteins. The role of ATP hydrolysis. J Biol Chem. 1991;266:1830–40. 10.1016/S0021-9258(18)52369-3. 1988449

[10] Yao N, O’Donnell M. Bacterial and eukaryotic replisome machines. JSM Biochem Mol Biol. 2016;3:1013. 28042596 PMC5199024

[11] Wang CC, Yeh LS, Karam JD. Modular organization of T4 DNA polymerase: evidence from phylogenetics. J Biol Chem. 1995;270:26558–64. 10.1074/jbc.270.44.26558. 7592876

[12] Wang J, Yu P, Lin TC et al. Crystal structures of an NH2-terminal fragment of T4 DNA polymerase and its complexes with single-stranded DNA and with divalent metal ions. Biochemistry. 1996;35:8110–9. 10.1021/bi960178r. 8679562

[13] Xia S, Konigsberg WH. RB69 DNA polymerase structure, kinetics, and fidelity. Biochemistry. 2014;53:2752–67. 10.1021/bi4014215. 24720884 PMC4018061

[14] Shamoo Y, Steitz TA. Building a replisome from interacting pieces: sliding clamp complexed to a peptide from DNA polymerase and a polymerase editing complex. Cell. 1999;99:155–66. 10.1016/S0092-8674(00)81647-5. 10535734

[15] Franklin MC, Wang J, Steitz TA. Structure of the replicating complex of a pol a family DNA polymerase. Cell. 2001;105:657–67. 10.1016/S0092-8674(01)00367-1. 11389835

[16] Berdis AJ, Soumillion P, Benkovic SJ. The carboxyl terminus of the bacteriophage T4 DNA polymerase is required for holoenzyme complex formation. Proc Natl Acad Sci USA. 1996;93:12822–7. 8917503 10.1073/pnas.93.23.12822 PMC24004

[17] Dalrymple BP, Kongsuwan K, Wijffels G et al. A universal protein–protein interaction motif in the eubacterial DNA replication and repair systems. Proc Natl Acad Sci USA. 2001;98:11627–32. 11573000 10.1073/pnas.191384398 PMC58780

[18] Shi K, Bohl TE, Park J et al. T4 DNA ligase structure reveals a prototypical ATP-dependent ligase with a unique mode of sliding clamp interaction. Nucleic Acids Res. 2018;46:10474–88. 10.1093/nar/gky776. 30169742 PMC6212786

[19] Shi J, Wen A, Jin S et al. Transcription activation by a sliding clamp. Nat Commun. 2021;12:1131. 10.1038/s41467-021-21392-0. 33602900 PMC7892883

[20] Moldovan G-L, Pfander B, Jentsch S. PCNA, the maestro of the replication fork. Cell. 2007;129:665–79. 10.1016/j.cell.2007.05.003. 17512402

[21] Moarefi I, Jeruzalmi D, Turner J et al. Crystal structure of the DNA polymerase processivity factor of T4 bacteriophage. J Mol Biol. 2000;296:1215–23. 10.1006/jmbi.1999.3511. 10698628

[22] Kong XP, Onrust R, Odonnell M et al. 3-Dimensional structure of the beta-subunit of *Escherichia coli* DNA Polymerase-Iii Holoenzyme - a Sliding DNA Clamp. Cell. 1992;69:425–37. 10.1016/0092-8674(92)90445-I. 1349852

[23] Krishna TSR, Kong X-P, Gary S et al. Crystal structure of the eukaryotic DNA polymerase processivity factor PCNA. Cell. 1994;79:1233–43. 10.1016/0092-8674(94)90014-0. 8001157

[24] Warbrick E . The puzzle of PCNA’s many partners. Bioessays. 2000;22:997–1006. 10.1002/1521-1878(200011)22:11<997::AID-BIES6>3.0.CO;2-#. 11056476

[25] Georgescu RE, Kim SS, Yurieva O et al. Structure of a sliding clamp on DNA. Cell. 2008;132:43–54. 10.1016/j.cell.2007.11.045. 18191219 PMC2443641

[26] De March M, Merino N, Barrera-Vilarmau S et al. Structural basis of human PCNA sliding on DNA. Nat Commun. 2017;8:13935. 10.1038/ncomms13935. 28071730 PMC5234079

[27] Lancey C, Tehseen M, Raducanu V-S et al. Structure of the processive human Pol δ holoenzyme. Nat Commun. 2020;11:1109. 10.1038/s41467-020-14898-6. 32111820 PMC7048817

[28] Zheng FW, Georgescu RE, Li H et al. Structure of eukaryotic DNA polymerase δ bound to the PCNA clamp while encircling DNA. Proc Natl Acad Sci USA. 2020;117:30344–53. 33203675 10.1073/pnas.2017637117 PMC7720213

[29] Li H, Zheng FW, O’Donnell M. Water skating: how polymerase sliding clamps move on DNA. FEBS J. 2021;288:7256–62. 10.1111/febs.15740. 33523561 PMC8325712

[30] Schrock RD, Alberts B. Processivity of the gene 41 DNA helicase at the bacteriophage T4 DNA replication fork. J Biol Chem. 1996;271:16678–82. 10.1074/jbc.271.28.16678. 8663273

[31] Alberts BM, Barry J, Bedinger P et al. Studies on DNA replication in the bacteriophage T4 *in vitro* system. Cold Spring Harbor Symp Quant Biol. 1983;47:655–68. 10.1101/SQB.1983.047.01.077. 6305581

[32] Kadyrov FA, Drake JW. Conditional coupling of leading-strand and lagging-strand DNA synthesis at bacteriophage T4 replication forks. J Biol Chem. 2001;276:29559–66. 10.1074/jbc.M101310200. 11390383

[33] Yang J, Zhuang Z, Roccasecca RM et al. The dynamic processivity of the T4 DNA polymerase during replication. Proc Natl Acad Sci USA. 2004;101:8289–94. 10.1073/pnas.0402625101. 15148377 PMC420387

[34] Nossal NG . DNA replication with bacteriophage T4 proteins. Purification of the proteins encoded by T4 genes 41, 45, 44, and 62 using a complementation assay. J Biol Chem. 1979;254:6026–31. 10.1016/S0021-9258(18)50514-7. 376524

[35] Frey MW, Nossal NG, Capson TL et al. Construction and characterization of a bacteriophage T4 DNA polymerase deficient in 3′→5′ exonuclease activity. Proc Natl Acad Sci USA. 1993;90:2579–83. 8464864 10.1073/pnas.90.7.2579 PMC46138

[36] Mastronarde DN . Automated electron microscope tomography using robust prediction of specimen movements. J Struct Biol. 2005;152:36–51. 10.1016/j.jsb.2005.07.007. 16182563

[37] Punjani A, Rubinstein JL, Fleet DJ et al. cryoSPARC: algorithms for rapid unsupervised cryo-EM structure determination. Nat Methods. 2017;14:290–6. 10.1038/nmeth.4169. 28165473

[38] Bepler T, Morin A, Rapp M et al. Positive-unlabeled convolutional neural networks for particle picking in cryo-electron micrographs. Nat Methods. 2019;16:1153–60. 10.1038/s41592-019-0575-8. 31591578 PMC6858545

[39] Goddard TD, Huang CC, Meng EC et al. UCSF ChimeraX: meeting modern challenges in visualization and analysis. Protein Sci. 2018;27:14–25. 10.1002/pro.3235. 28710774 PMC5734306

[40] Brown A, Long F, Nicholls RA et al. Tools for macromolecular model building and refinement into electron cryo-microscopy reconstructions. Acta Crystallogr D Biol Crystallogr. 2015;71:136–53. 10.1107/S1399004714021683. 25615868 PMC4304694

[41] Adams PD, Afonine PV, Bunkóczi G et al. PHENIX: a comprehensive Python-based system for macromolecular structure solution. Acta Crystallogr D Biol Crystallogr. 2010;66:213–21. 10.1107/S0907444909052925. 20124702 PMC2815670

[42] Chen VB, Arendall WB, Headd JJ et al. MolProbity: all-atom structure validation for macromolecular crystallography. Acta Crystallogr D Biol Crystallogr. 2010;66:12–21. 10.1107/S0907444909042073. 20057044 PMC2803126

[43] Williams CJ, Headd JJ, Moriarty NW et al. MolProbity: more and better reference data for improved all-atom structure validation. Protein Sci. 2018;27:293–315. 10.1002/pro.3330. 29067766 PMC5734394

[44] Robert X, Gouet P. Deciphering key features in protein structures with the new ENDscript server. Nucleic Acids Res. 2014;42:W320–4. 10.1093/nar/gku316. 24753421 PMC4086106

[45] Ashkenazy H, Abadi S, Martz E et al. ConSurf 2016: an improved methodology to estimate and visualize evolutionary conservation in macromolecules. Nucleic Acids Res. 2016;44:W344–50. 10.1093/nar/gkw408. 27166375 PMC4987940

[46] Kaboord BF, Benkovic SJ. Accessory proteins function as matchmakers in the assembly of the T4 DNA polymerase holoenzyme. Curr Biol. 1995;5:149–57. 10.1016/S0960-9822(95)00036-4. 7743178

[47] Yao N, Turner J, Kelman Z et al. Clamp loading, unloading and intrinsic stability of the PCNA, beta and gp45 sliding clamps of human, *E. coli* and T4 replicases. Genes Cells. 1996;1:101–13. 10.1046/j.1365-2443.1996.07007.x. 9078370

[48] Park J, Youn HS, An JY et al. Structure of new binary and ternary DNA polymerase complexes from bacteriophage RB69. Front Mol Biosci. 2021;8:704813. 10.3389/fmolb.2021.704813. 34869578 PMC8639217

[49] Ren Z . Molecular events during translocation and proofreading extracted from 200 static structures of DNA polymerase. Nucleic Acids Res. 2016;44:7457–74. 27325739 10.1093/nar/gkw555 PMC5009745

[50] Salinas F, Benkovic SJ. Characterization of bacteriophage T4-coordinated leading- and lagging-strand synthesis on a minicircle substrate. Proc Natl Acad Sci USA. 2000;97:7196–201. 10.1073/pnas.97.13.7196. 10860983 PMC16522

[51] Trakselis MA, Roccasecca RM, Yang J et al. Dissociative properties of the proteins within the bacteriophage T4 replisome. J Biol Chem. 2003;278:49839–49. 10.1074/jbc.M307405200. 14500719

[52] Chen D, Yue H, Spiering MM et al. Insights into Okazaki fragment synthesis by the T4 replisome: the fate of lagging-strand holoenzyme components and their influence on Okazaki fragment size. J Biol Chem. 2013;288:20807–16. 10.1074/jbc.M113.485961. 23729670 PMC3774352

[53] Yang J, Trakselis MA, Roccasecca RM et al. The application of a minicircle substrate in the study of the coordinated T4 DNA replication. J Biol Chem. 2003;278:49828–38. 10.1074/jbc.M307406200. 14500718

[54] Johnson DE, Takahashi M, Hamdan SM et al. Exchange of DNA polymerases at the replication fork of bacteriophage T7. Proc Natl Acad Sci USA. 2007;104:5312–7. 17369350 10.1073/pnas.0701062104 PMC1838503

[55] Hamdan SM, Johnson DE, Tanner NA et al. Dynamic DNA helicase–DNA polymerase interactions assure processive replication fork movement. Mol Cell. 2007;27:539–49. 10.1016/j.molcel.2007.06.020. 17707227

[56] Hamdan SM, Marintcheva B, Cook T et al. A unique loop in T7 DNA polymerase mediates the binding of helicase-primase, DNA binding protein, and processivity factor. Proc Natl Acad Sci USA. 2005;102:5096–101. 15795374 10.1073/pnas.0501637102 PMC556000

[57] Lee SJ, Marintcheva B, Hamdan SM et al. The C-terminal residues of bacteriophage T7 gene 4 helicase-primase coordinate helicase and DNA polymerase activities. J Biol Chem. 2006;281:25841–9. 10.1074/jbc.M604602200. 16807231

[58] Loparo JJ, Kulczyk AW, Richardson CC et al. Simultaneous single-molecule measurements of phage T7 replisome composition and function reveal the mechanism of polymerase exchange. Proc Natl Acad Sci USA. 2011;108:3584–9. 21245349 10.1073/pnas.1018824108 PMC3048139

[59] Geertsema HJ, Kulczyk AW, Richardson CC et al. Single-molecule studies of polymerase dynamics and stoichiometry at the bacteriophage T7 replication machinery. Proc Natl Acad Sci USA. 2014;111:4073–8. 24591606 10.1073/pnas.1402010111 PMC3964090

[60] Wallen JR, Zhang H, Weis C et al. Hybrid methods reveal multiple flexibly linked DNA polymerases within the bacteriophage T7 replisome. Structure. 2017;25:157–66. 10.1016/j.str.2016.11.019. 28052235 PMC5267931

[61] McInerney P, Johnson A, Katz F et al. Characterization of a triple DNA polymerase replisome. Mol Cell. 2007;27:527–38. 10.1016/j.molcel.2007.06.019. 17707226

[62] Georgescu RE, Kurth I, O’Donnell ME. Single-molecule studies reveal the function of a third polymerase in the replisome. Nat Struct Mol Biol. 2012;19:113–6. 10.1038/nsmb.2179. PMC3721970 22157955

[63] Dohrmann PR, Correa R, Frisch RL et al. The DNA polymerase III holoenzyme contains γ and is not a trimeric polymerase. Nucleic Acids Res. 2016;44:1285–97. 10.1093/nar/gkv1510. 26786318 PMC4756838

[64] López de Saro FJ, O’Donnell M. Interaction of the β sliding clamp with MutS, ligase, and DNA polymerase I. Proc Natl Acad Sci USA. 2001;98:8376–80. 11459978 10.1073/pnas.121009498 PMC37446

[65] Fuchs RP, Fujii S. Translesion synthesis in *Escherichia coli*: lessons from the NarI mutation hot spot. DNA Repair (Amst). 2007;6:1032–41. 10.1016/j.dnarep.2007.02.021. 17403618

[66] Zhao L, Washington MT. Translesion synthesis: insights into the selection and switching of DNA polymerases. Genes. 2017;8:24. 10.3390/genes8010024. 28075396 PMC5295019

[67] Bunting KA, Roe SM, Pearl LH. Structural basis for recruitment of translesion DNA polymerase Pol IV/DinB to the beta-clamp. EMBO J. 2003;22:5883–92. 10.1093/emboj/cdg568. 14592985 PMC275425

[68] Indiani C, McInerney P, Georgescu R et al. A sliding-clamp toolbelt binds high- and low-fidelity DNA polymerases simultaneously. Mol Cell. 2005;19:805–15. 10.1016/j.molcel.2005.08.011. 16168375

[69] Lewis JS, Spenkelink LM, Schauer GD et al. Tunability of DNA polymerase stability during eukaryotic DNA replication. Mol Cell. 2020;77:17–25.e5. 10.1016/j.molcel.2019.10.005. 31704183 PMC6943181

[70] Lynch L, Chistol G. Eukaryotic lagging strand synthesis is distributive. bioRxiv, 10.1101/2025.09.17.676973, 18 September 2025, preprint: not peer reviewed.

