# Supplementary material for: Structural insights into the exchange mechanism of a replicative DNA polymerase
Source: Nucleic Acids Res. 2025 Dec 29;53(22):gkaf1359. doi: 10.1093/nar/gkaf1359 (PMC12746103; doi:10.1093/nar/gkaf1359)
Supplement: gkaf1359_Supplemental_File [file gkaf1359_supplemental_file.pdf]

Supplementary information for

Structural insights into the exchange mechanism of a replicative DNA polymerase

by Feng et al.

This document contains

- 1 Supplementary table
- 9 Supplementary figures

Supplementary Table 1. Cryo-EM data collection, refinement, and validation statistics for the T4 one- and two-polymerase complexes.

	State 1 Two-Pol complex (EMDB-47531) (PDB 9E5Y)	State 2 Two-Pol complex (EMDB-47824) (PDB 9EA2)	State 3 Two-Pol complex (EMDB-47826) (PDB 9EA6)	T4 holoenzyme One-Pol mutant complex (EMDB-47825) (PDB 9EA3)
**Data collection and processing**				
Magnification	105,000	105,000	105,000	105,000
Voltage (kV)	300	300	300	300
Electron exposure (e–/Å^2)	60	60	60	60
Defocus range (μm)	1.0 to 2.0	1.0 to 2.0	1.0 to 2.0	1.0 to 2.0
Pixel size (Å)	0.828	0.828	0.828	0.828
Symmetry imposed	C1	C1	C1	C1
Initial particle images (no.)	922,085	922,085	922,085	772,599
Final particle images (no.)	349,355	164,340	133,535	372,199
Map resolution (Å)	3.57	3.7	3.74	3.99
FSC threshold	0.143	0.143	0.143	0.143
Map resolution range (Å)	2.0-10.0	2.0-10.0	2.0-10.0	2.0-10.0
**Refinement**				
Initial model used (PDB code)	polymerase (AlphaFold), sliding clamp (1ZCD), and DNA (7KC0)			
Model resolution (Å)	3.9	4.06	4.1	4.24
FSC threshold	0.5	0.5	0.5	0.5
Model resolution range (Å)	2.0-10.0	2.0-10.0	2.0-7.0	2.0-10.0
Map sharpening *B* factor (Å^2)	147.6	139.0	138.0	194.3
Model composition				
Non-hydrogen atoms	20897	20933	21050	13679
Protein residues	2479	2482	2479	1582
Nucleotides	53	53	60	56
B factors (Å^2)				
Protein	95.9	157.60	72.64	145.90
Nucleotide	240.6	330.82	151.72	215.78
R.m.s. deviations				
Bond lengths (Å)	0.006	0.004	0.004	0.005
Bond angles (°)	1.003	1.000	0.966	0.909
Validation				
MolProbity score	2.12	1.99	1.95	2.56
Clashscore	17.33	16.89	15.766	19.27
Poor rotamers (%)	0.79	0.56	0.23	4.63
Ramachandran plot				
Favored (%)	94.45	96.15	96.23	96.0
Allowed (%)	5.55	3.85	3.77	4.0
Disallowed (%)	0.0	0.0	0.0	0.0

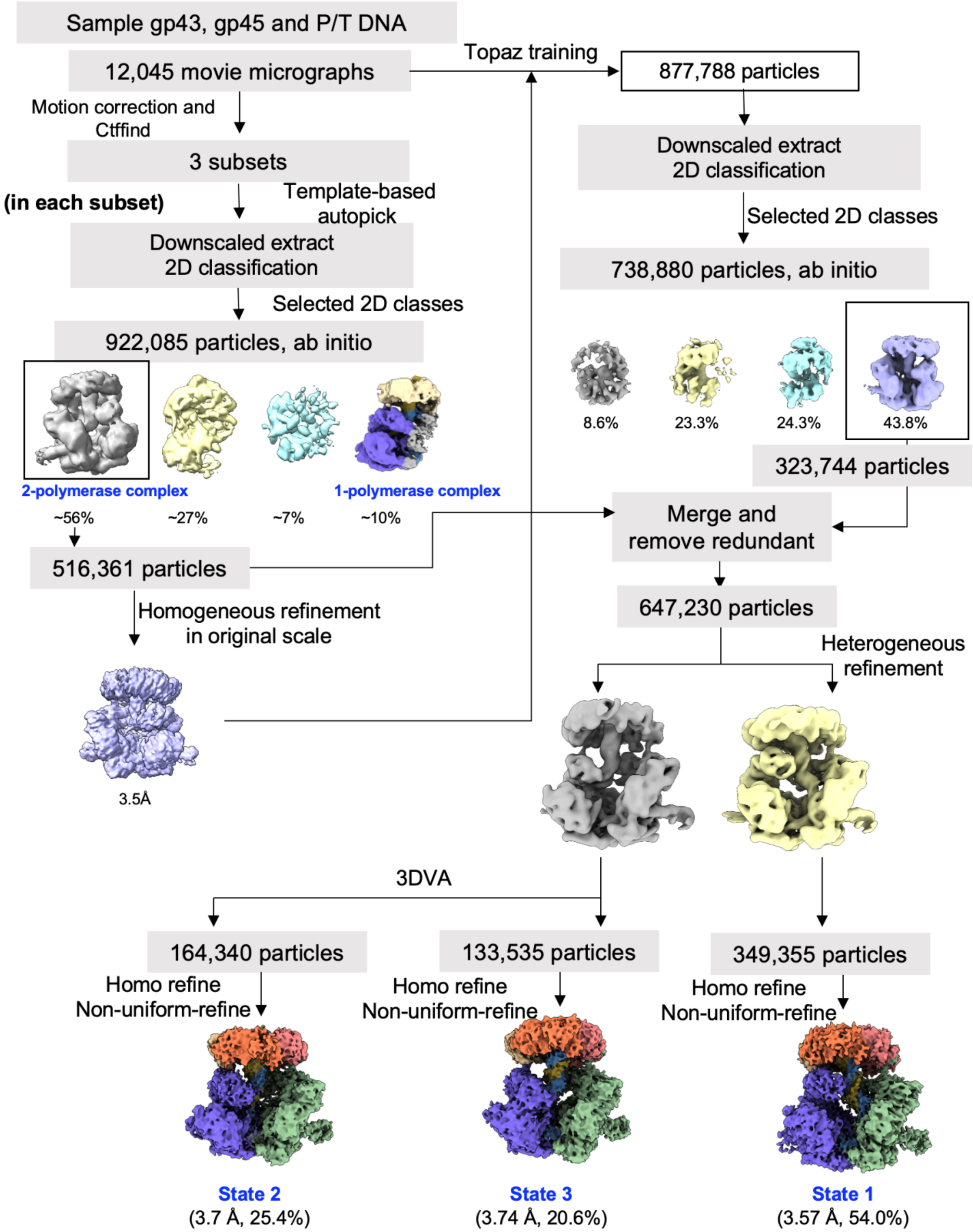

Supplementary Fig. 1. Workflow for cryo-EM data processing of T4 gp43(exo-) polymerase complexes. Mixtures containing gp43(exo-) polymerase, gp45 clamp, P/T DNA, and ddTTP yielded two distinct complex types: a one-polymerase complex and a two-polymerase complex. Under the cryo-EM imaging conditions (6 μM polymerase), the two-polymerase complex constituted the predominant species (90% of particles), while the one-polymerase complex represented a smaller subset (10%). Three discrete conformational states (1-3) were resolved within the two-polymerase complex dataset.

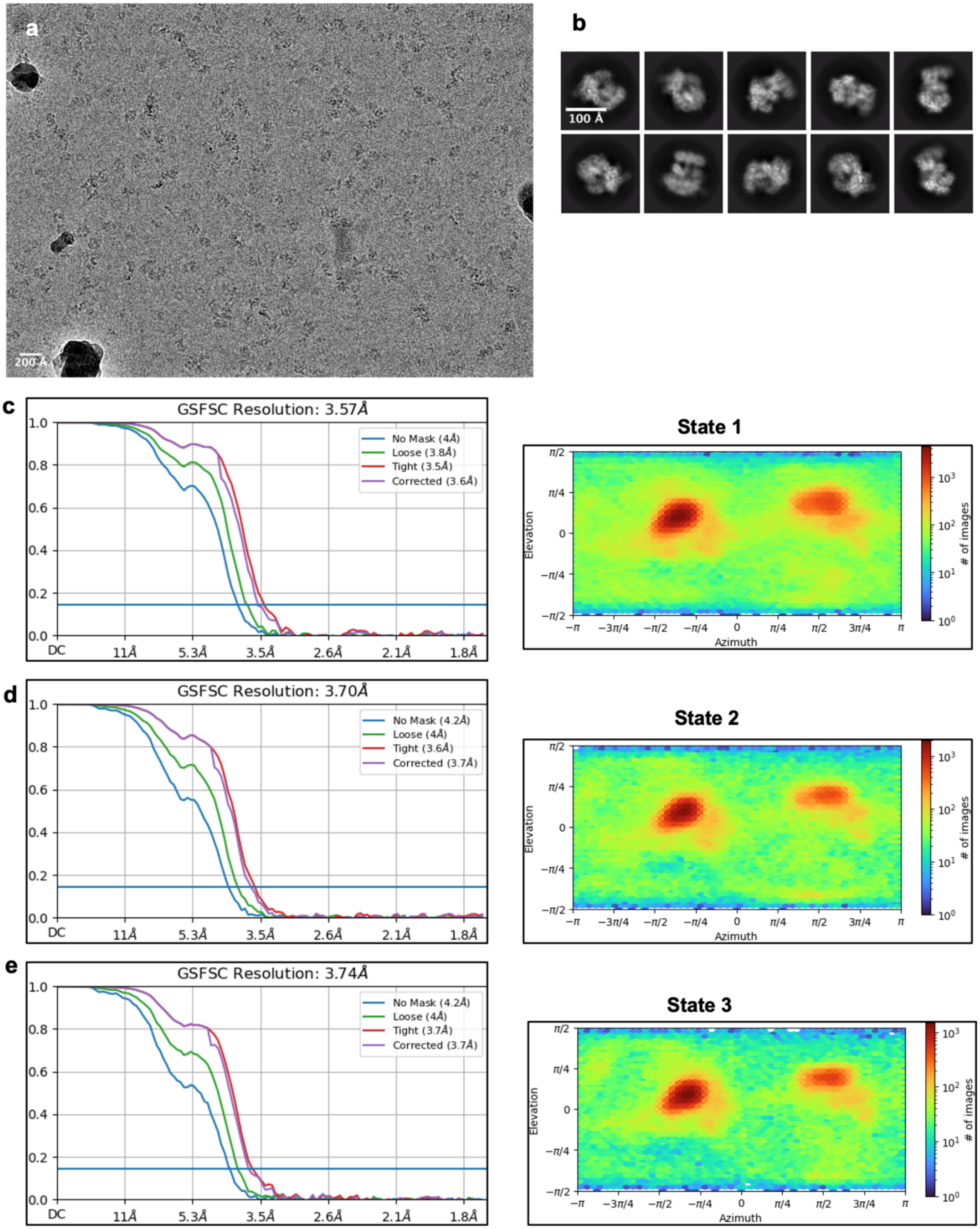

Supplementary Fig. 2. Cryo-EM data statistics for the three EM maps of the two-polymerase complex (states 1-3). a) Representative raw micrograph from a dataset comprising 12,045 recorded micrographs. **b**) Selected 2D class averages showing that most particles contain two gp43(exo-) molecules together with the clamp and DNA components. **c-e**) FSC curves (left) and angular distribution plots (right) for the two-polymerase complex in state 1 (**c**), state 2 (**d**), and state 3 (**e**).

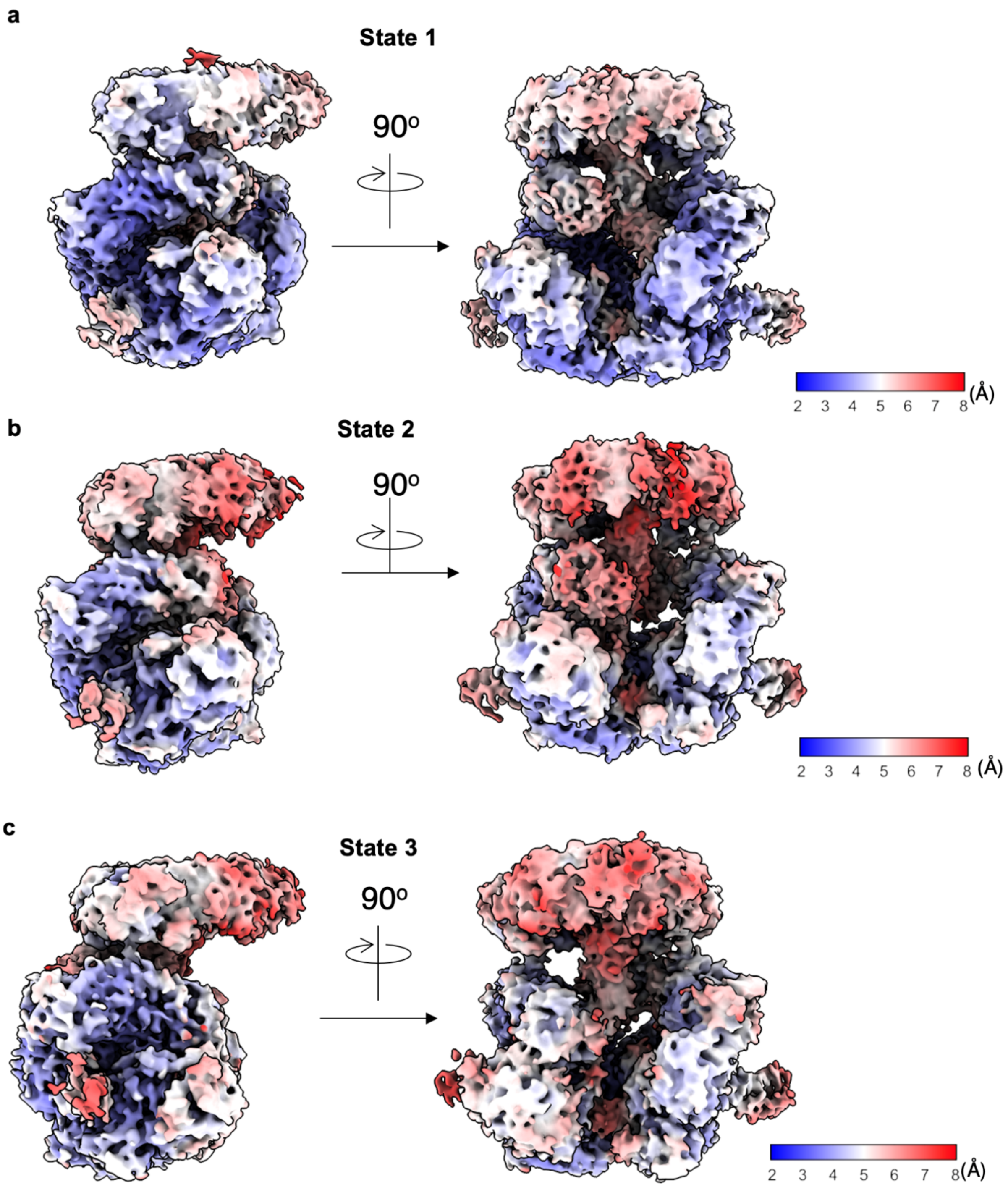

Supplementary Fig. 3. Local resolution estimation of cryo-EM 3D reconstructions for the three conformational states of the two-polymerase complex. Surface-rendered EM maps for (**a**), state 1; (**b**), state 2; and (**c**), state 3 are shown in side and front views. Color-coding indicates local resolution distribution within each reconstruction.

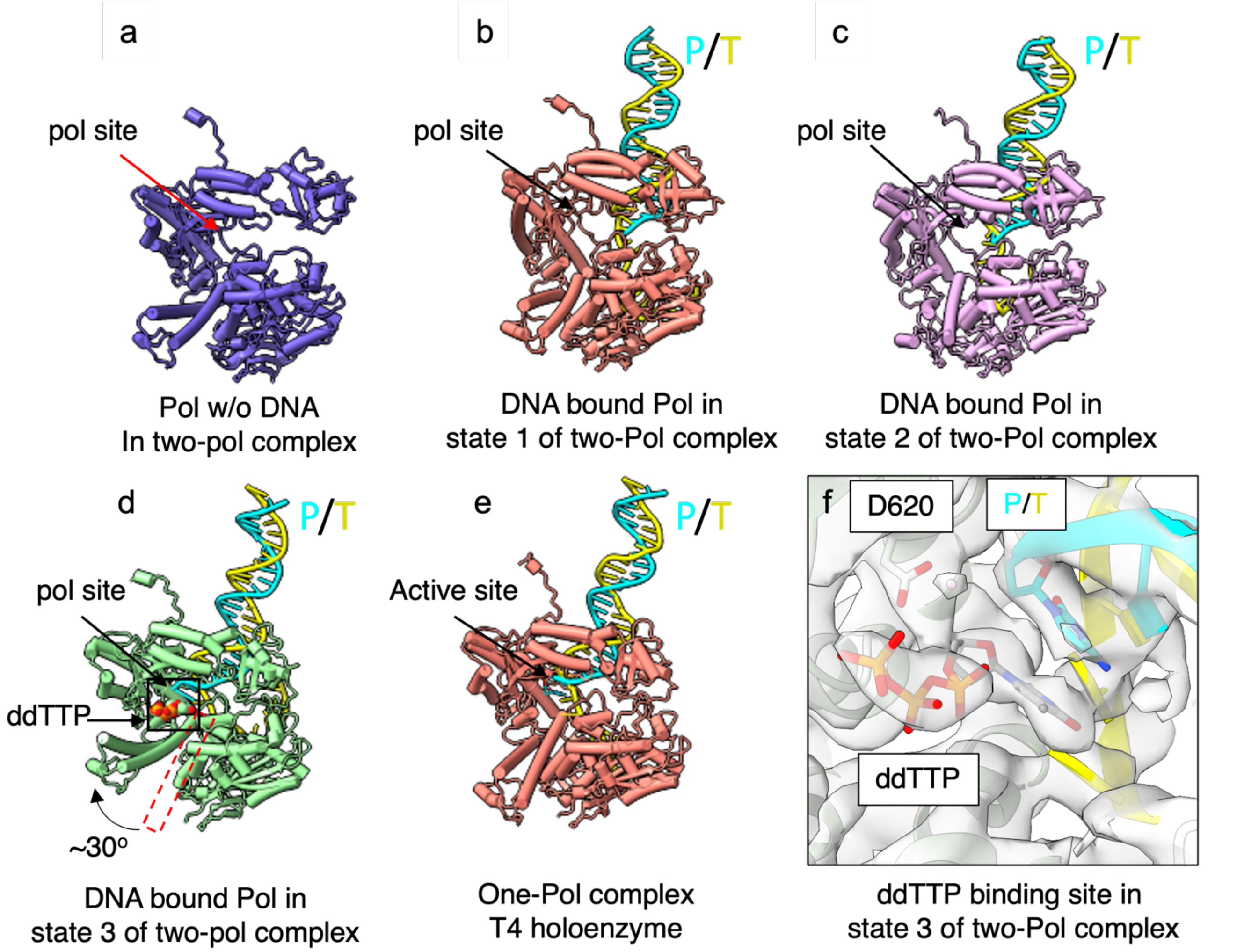

Supplementary Fig. 4. Polymerization active site conformations across T4 gp43(exo-) molecules characterized in this study. a) The three apo polymerases lacking DNA binding in all conformational states of the two-polymerase complex exhibit finger domains in the open configuration. **b, c**) In state 1 and state 2 of the two-polymerase complex, the primer 3' terminus does not reach the polymerization catalytic site (pol site). **d**) In state 3, the primer 3' terminus occupies the pol site, accompanied by finger-domain closure involving a ~30° upward movement that seals the active site. **e**) In the one-polymerase T4 holoenzyme, the primer 3' terminus is positioned at the pol site, while the finger domain remains open and poised for nucleotide binding. Polymerase structures in panels a–e were aligned, rendered in cartoon representation, and arranged using ChimeraX. **f**) EM density corresponding to the incoming nucleotide analog ddTTP in state 3 of the two-polymerase complex, shown as a surface-rendering contoured at σ = 4 and overlaid with the atomic model.

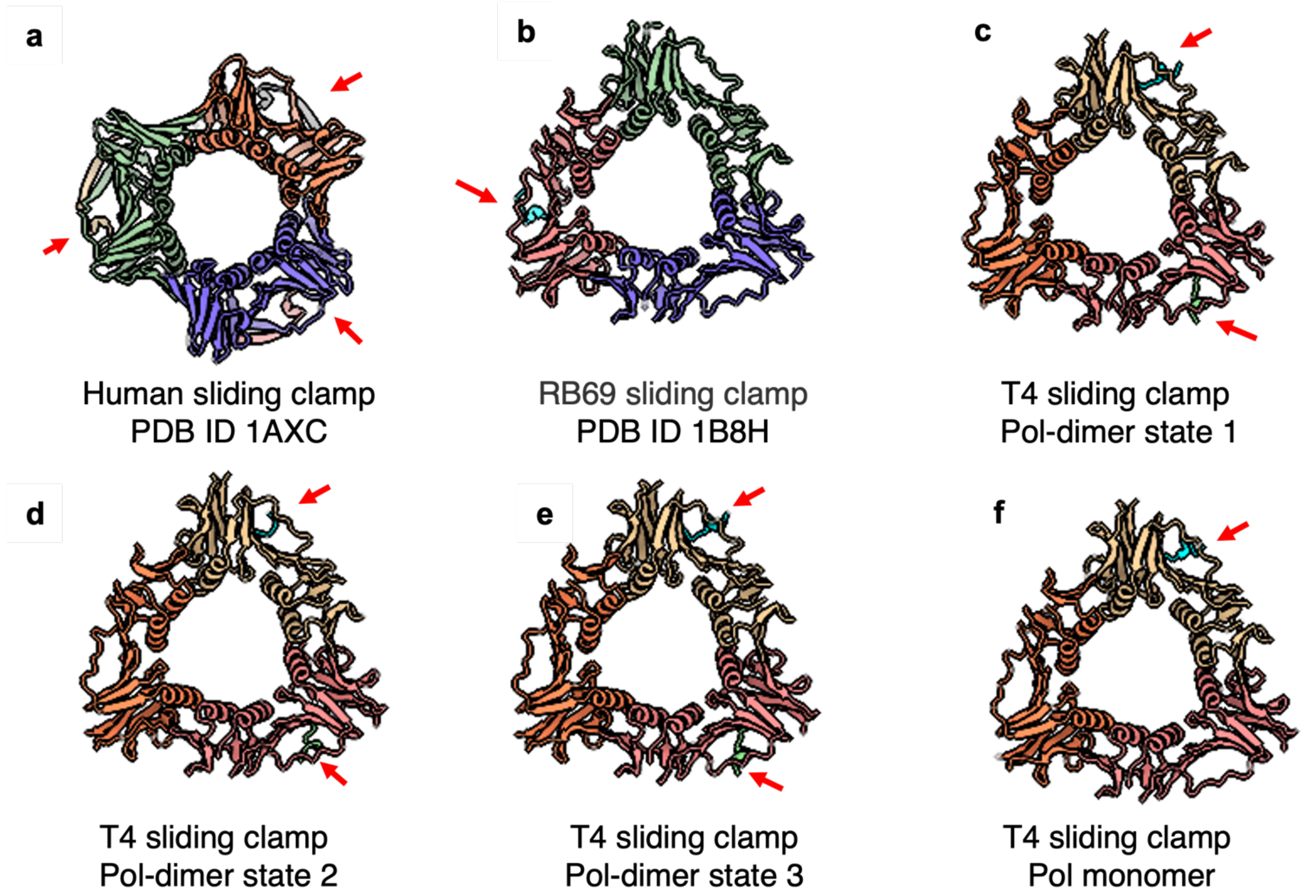

Supplementary Fig. 5. Structural comparison of sliding clamps. Top views of solved sliding clamps are shown **a**) human PCNA (PDB ID 1AXC), **b**) RB69 bacteriophage clamp (PDB ID 1B8H), **c-e**) T4 gp45 in two-polymerase states 1-3 (this study), and **f**) T4 gp45 in the one-polymerase state (this study). The human PCNA clamp exhibits a hexagonal or circular DNA channel, characteristic of cellular sliding clamps across bacteria, archaea, and eukaryotes. All clamp structures were aligned and arranged in a grid using ChimeraX. DNA duplexes were omitted for clarity. Arrows indicate the locations of clamp-interacting peptides (CIP) at their respective binding sites.

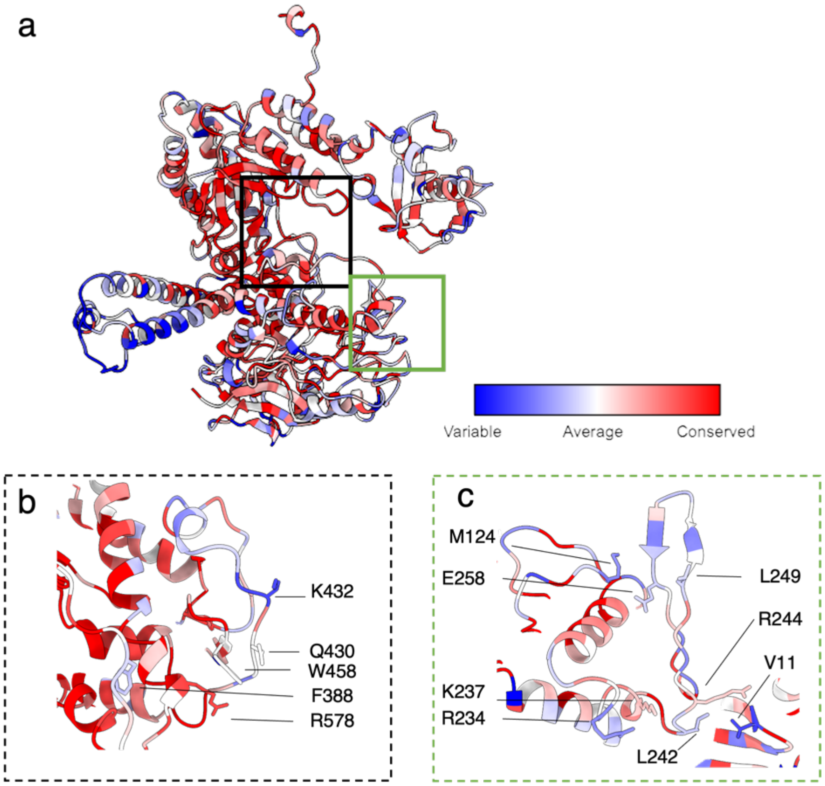

Supplementary Fig. 6. The dimerization interface of T4 gp43 polymerase is not well conserved among phages. (**a**) Structure of the gp43 polymerase shown in cartoon representation, color coded according to residue-level conservation scores calculated using ConSurf. (**b, c**) Close-up views of the two dimerization regions indicated by the black and green boxes in panel **a**. Dimerization residues are labeled and shown as sticks. The variable conservation of residues across these regions indicates that the dimer interface is not universally conserved among phage polymerases.

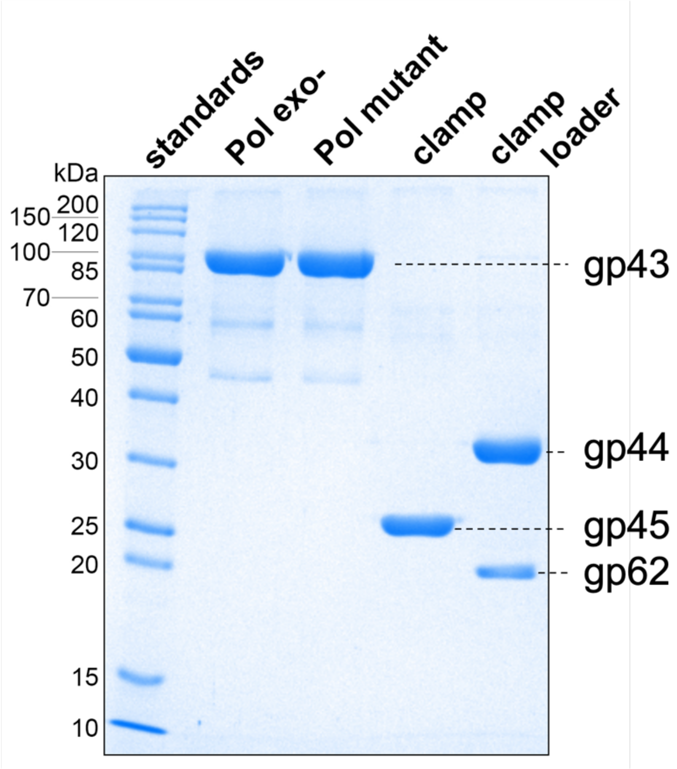

Supplementary Fig. 7. Purified protein quality assessment. SDS-PAGE analysis of individually purified proteins used for assembly of the T4 polymerase–clamp complex and for kinetic assays. "Pol exo-" denotes the exonuclease-deficient polymerase [gp43(exo-)], whereas "Pol mutant" refers to the exonuclease-deficient polymerase containing three dimer interface substitutions (D75R, Q430E, and K432E).

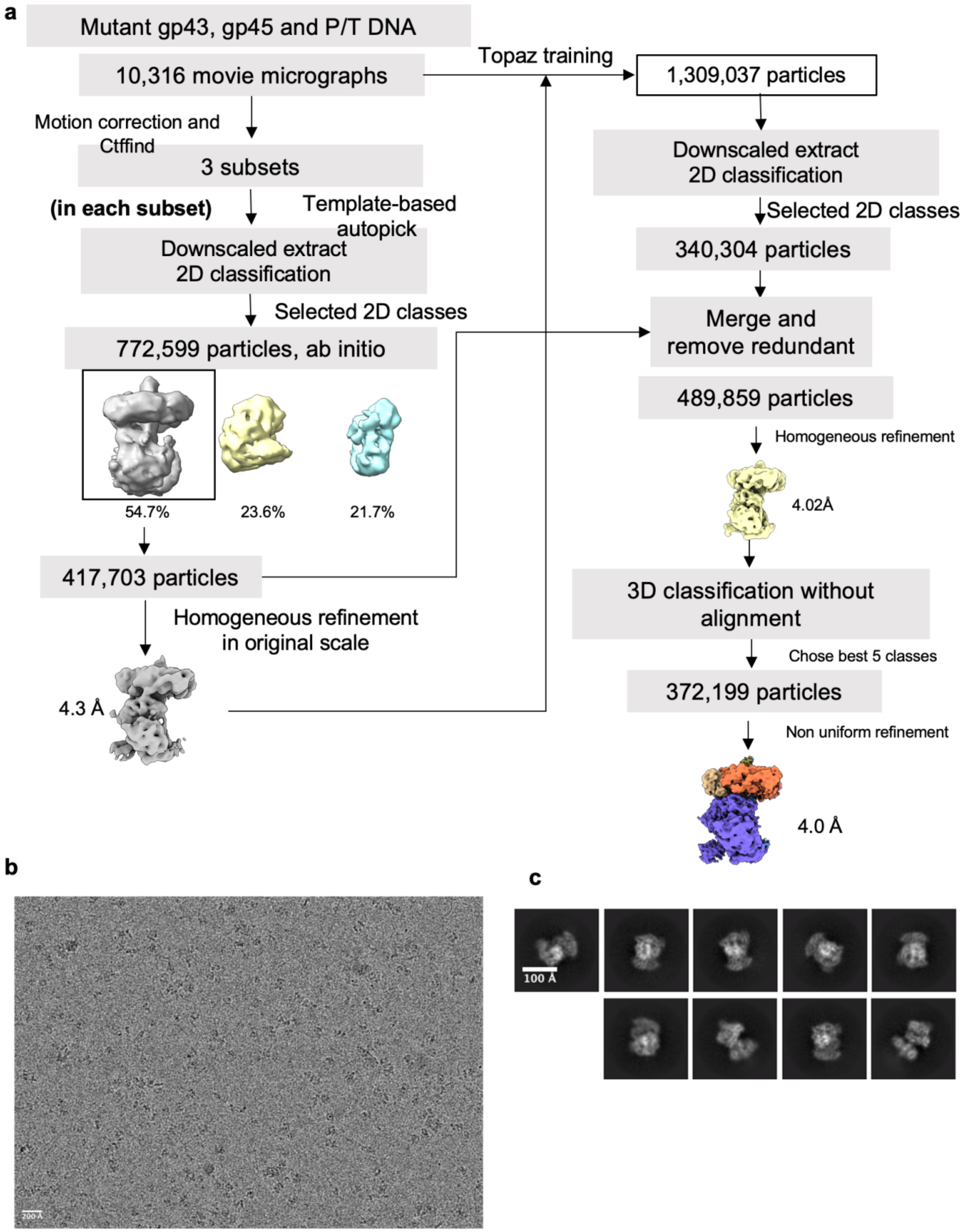

Supplementary Fig. 8. Cryo-EM image processing workflow for the one-polymerase T4 holoenzyme assembled with the dimer-interface mutant gp43(exo-) polymerase. a) Data processing workflow for cryo-EM images of in vitro mixtures containing the dimer-interface mutant gp43(exo-) polymerase, gp45 clamp, P/T DNA substrate, and ddTTP. Image processing yielded a 3D reconstruction of the T4 holoenzyme containing a single polymerase at an overall resolution of 4.0 Å. **b**) Representative raw micrograph from a dataset comprising 10,316 recorded micrographs. **c**) Selected 2D class averages show that all particle complexes contain only a single polymerase.

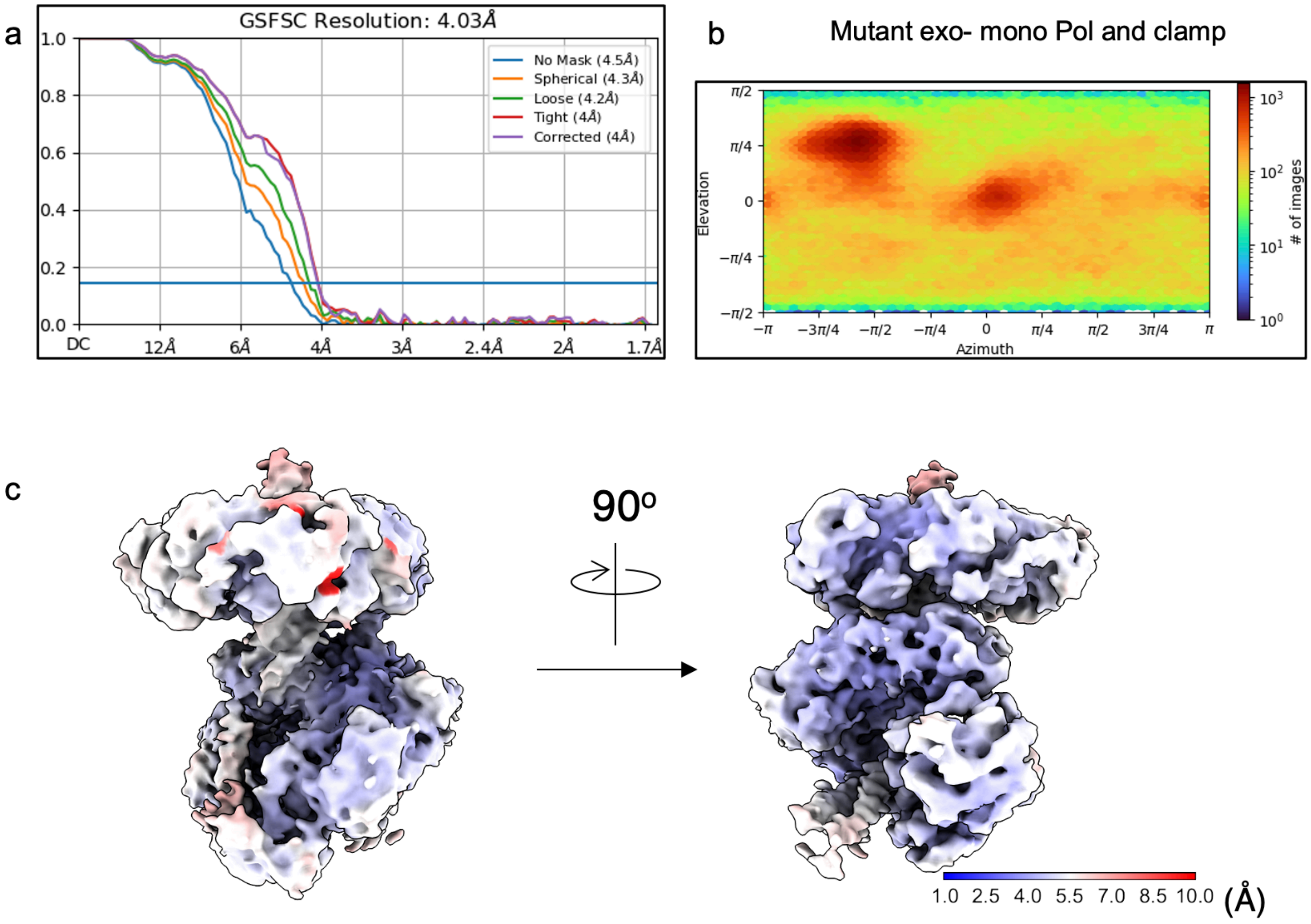

Supplementary Fig. 9. Resolution estimation for the EM map of T4 holoenzyme assembled with dimer-interface mutant gp43(exo-). a) Fourier shell correlation (FSC) analysis indicates an overall map resolution of 4.0 Å. **b**) Angular distribution analysis shows good coverage of particle orientations. **c**) Orthogonal views of the surface-rendered EM map, color-coded according to local resolution estimates.
